# Creating a suprazyme: integrating a molecular enzyme mimic with a nanozyme for enhanced catalysis†

**DOI:** 10.1039/d4sc04577g

**Published:** 2024-09-26

**Authors:** Pavlo Hyziuk, Matteo Flaibani, Paola Posocco, Volodymyr Sashuk

**Affiliations:** a Institute of Physical Chemistry, Polish Academy of Sciences Kasprzaka 44/52 01-224 Warsaw Poland vsashuk@ichf.edu.pl; b Department of Engineering and Architecture, University of Trieste Via Alfonso Valerio, n. 6/A 34127 Trieste Italy paola.posocco@dia.units.it

## Abstract

Enzyme mimics, due to their limited complexity, traditionally display low catalytic efficiency. Herein we present a strategy that enables the transformation of a slow-acting catalyst into a highly active one by creating a non-covalent suprastructure, termed “suprazyme”. We show that cucurbit[7]uril macrocycles, rudimentary molecular enzyme mimics, embedded within an anionic monolayer on the surface of gold nanoparticles, outperform individual cucurbit[7]urils as well as nanoparticles, which also exhibit catalytic enzyme-like activity and thus act as nanozymes, by over 50 times, showcasing a 1044-fold acceleration in a model oxime formation reaction. The superior performance of such a suprazyme is attributed to a synergistic interplay between the organic monolayer and macrocycles, which is accompanied by a decreased local polarity and pH that favors the acid-catalyzed condensation process. The proposed approach holds promise for developing diverse suprazymes, contingent upon achieving a complementary structure and mechanism of action between the molecular catalyst and nanoparticles.

## Abstract 2

We present a supramolecular assembly of cucurbituril macrocycles and organomodified gold nanoparticles, referred to as “suprazyme”, with catalytic activity surpassing its components by over 50 times in a model oximation reaction.

## Graphical abstract

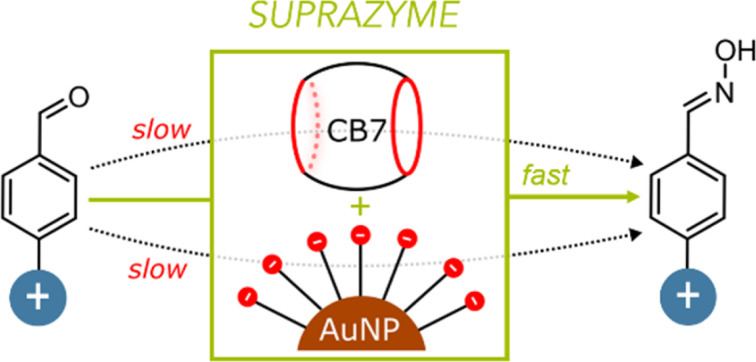

## Introduction

Enzymes are intricate protein molecules that catalyze chemical reactions in living cells with extraordinary efficiency. However, the application of many of them outside the cellular environment is significantly hampered due to high cost, low stability, and narrow substrate and reaction scope. These limitations have spurred ongoing efforts to discover synthetic analogs of these natural catalysts that are simpler, more cost-effective, stable, and versatile. Among readily accessible molecules, macrocyclic compounds with a deep inner cavity, known as cavitands, bear structural resemblance to enzymes and have historically served as their mimics.^1,2^ Unfortunately, unfunctionalized cavitands have proven inefficient in catalysis,^3^ because of the lack of an ancillary protein-like backbone, a satisfactory substrate–cavity match, and precisely oriented catalytic residues, the selective introduction of which into their symmetrical structure is a difficult synthetic task. This prompted us to search for alternative means to enhance their catalytic performance.

Our focus turned to inorganic nanoparticles coated with organic ligands, which, due to the cooperativity between functional groups,^4–8^ can also exhibit enzyme-like behavior and are therefore often called nanozymes.^9,10^ These fascinating nanoentities intrigued us by their increased catalytic activity upon the adsorption of biomolecules such as peptides^11^ and DNA^12^ onto their surfaces and the possibility to create – within the organic monolayer – molecular environments that are distinct from bulk surroundings by simply changing ligand chemistry.^13–17^ In particular, we aimed at exploring the impact of internal voids, which frequently occur in their ligand shells due to surface curvature and ligand clustering^13,14,18^ and may therefore resemble binding pockets of proteins, on catalytic activity with the prospect of utilizing them for multivalent interactions with macrocycles. This reasoning has ultimately led us to the construction of what we term a “suprazyme”, a fully artificial enzyme mimic that is a supramolecular ensemble of a macrocycle acting as a molecular enzyme mimic and an organic-coated metal nanoparticle functioning as a nanozyme that is catalytically far more effective than each individual constituent of the system. This new catalytic design is fundamentally distinct from other suprazymes,^19,20^ and previous macrocycle–nanoparticle hybrids,^21^ where either a molecular or nanoscale component served as a catalyst, opening the way for the improvement of molecular catalysts without resorting to covalent modification, using a functional organic monolayer as a non-covalent, adaptable and easily reconfigurable ligand shell.

## Results and discussion

**Fig. 1:**
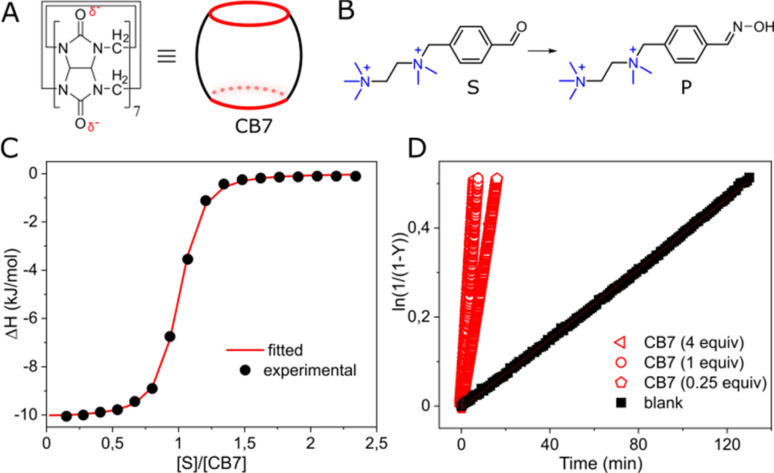
(A) Chemical structure and cartoon representation of cucurbit[7]uril (CB7); (B) oxime formation reaction; (C) titration of CB7 with the substrate, water, pH = 6 (adjusted with HCl/NaOH), 298 K; (D) kinetic traces for the reaction progress at various amounts of CB7, [*S*] = 0.01 mM, water, pH = 6 (adjusted with HCl/NaOH), 298 K.

We investigated cucurbit[7]uril (CB7), a well-known supramolecular enzyme-mimicking catalyst^22–28^ but challenging macrocycle when it comes to functionalization^29,30^ (Fig. 1A). As a model reaction, we studied oxime formation, a common (bio)conjugation tool,^31^ utilizing hydroxylamine and an aromatic aldehyde (Fig. 1B). Hydroxylamine was used in an excess (20 equiv.) to buffer the aqueous solution at pH = 6,‡‡The p*K*_a_ of hydroxylamine is 5.94, so it has the greatest buffering capacity at pH close to this value. maintaining the pseudo-first-order kinetics and shifting the equilibrium toward product formation with only ≈5% of the hydrated aldehyde remaining unreacted (Fig. S3, ESI†). The aldehyde (substrate) was designed so that the aldehyde group in the expected CB7–substrate complex (Fig. S22, ESI†) is positioned in proximity to one of the carbonyl rims, allowing for the electrostatic stabilization of the forming protonated intermediates.^32^ The other part of the aldehyde molecule was equipped with a doubly cationic group to ensure strong binding with CB7 *via* charge–dipole interactions with the opposite carbonyl rim. NMR analysis confirmed the intracavitary location of the substrate (Fig. S13, ESI†), and isothermal titration calorimetry (ITC) corroborated a 1 : 1 interaction, establishing an association constant (*K*_a_) of 1.40 ± 0.10 × 10^6^ M^−1^ (Fig. 1C). Indeed, when we added CB7 to the mixture of aldehyde and hydroxylamine, the reaction sped up. However, the acceleration, defined as *k*_obs_/*k*_w_, where *k*_obs_ and *k*_w_ are reaction rates with and without CB7, like in other condensation reactions promoted by CB7,^33–35^ was relatively modest: approximately 17-fold when an equimolar amount (1 equiv.) of the macrocycle was used and about 8-fold for a substoichiometric loading (0.25 equiv.), reaffirming the role of CB7 as a catalyst (Fig. 1D).

**Fig. 2:**
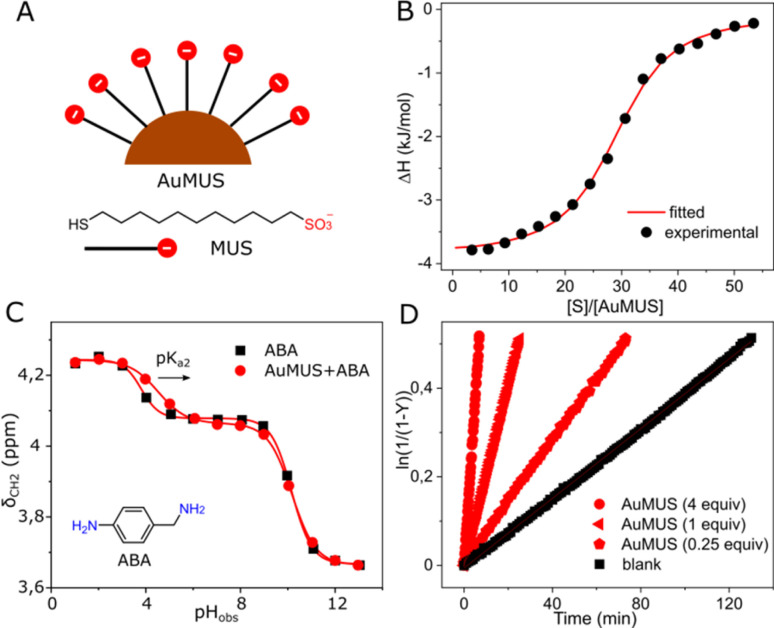
(A) Chemical structure and cartoon representation of anionic ligands and gold nanoparticles covered by them; (B) titration of AuMUS with the substrate, water, pH = 6 (adjusted with HCl/NaOH), 298 K; (C) titration of AuMUS with 4-aminobenzylamine, water, 298 K; (D) kinetic traces for the reaction progress at various amounts of AuMUS, [*S*] = 0.01 mM, water, pH = 6 (adjusted with HCl/NaOH), 298 K.

To increase the catalytic efficiency of CB7, we sought a cocatalyst with a similar function, specifically one that facilitates protonation. We synthesized gold nanoparticles (AuNPs) with a metal core of approximately 2.5 nm in diameter, coated with 11-mercapto-1-undecanesulfonate (MUS) ligands (Fig. 2A), with the capacity to form a robust binding interaction with the doubly positively charged substrate. This anticipation was verified through the ^1^H NMR spectrum, showing line broadening (Fig. S13, ESI†), MD and ITC measurements, revealing *K*_a_ = 2.05 ± 0.26 × 10^5^ M^−1^ and an average occupancy of approximately 29 substrate molecules per a single NP (AuMUS, Fig. 2B and S23, ESI†).

In addition, we hypothesized that the anionic coating should afford a local acidic environment on the nanoparticle surface by analogy with cationic groups, which cause the opposite effect.^11^ To prove this supposition, we measured the acidity of 4-aminobenzylamine (ABA), a pH-sensitive probe with a structure and charge reminiscent of the substrate under reaction conditions in the presence of nanoparticles (Fig. 2C). We found that while the apparent p*K*_a1_ remains almost unchanged, presumably due to a weak binding of the neutral probe to the negatively charged organic monolayer, the p*K*_a2_ experienced a substantial increase (by 0.7 units), indicating binding of the monoprotonated probe followed by enhanced protonation of the second amine group, supporting our hypothesis.

After that, we tested the MUS-coated AuNPs in catalysis. Similar to CB7, we observed an enhancement in the oxime product formation. However, the efficacy of the NPs was notably less pronounced with approximately 5 and 2-fold accelerations for 1 and 0.25 equiv. of adsorbed MUS ligands, the amount of which was assessed from thermogravimetric analysis (Fig. S8, ESI†), respectively (Fig. 2D). The observed effect is probably related to the weaker binding of the substrate to the monolayer (Tables S2 and S4, ESI†) and smaller p*K*_a_ changes induced by the NPs compared to CB7. For the macrocycle, the binding strength and local p*K*_a_ changes, that are one of the hallmarks of enzyme-like catalysis^10^ (1.3 and 2.2 units, as determined from NMR titration, Fig. S12, ESI†), are markedly larger, rendering it a more effective active component of the system.

In a parallel set of experiments, we examined AuNPs coated with positively charged (TMA, a thiol terminated with an ammonium group) and neutral (PEG, a thiol comprising an oligo(ethylene glycol) chain) ligands, given that the former might interact with CB7 (ref. ^36^) and the latter with cationic groups^37^ of the substrate. We also performed the reaction in the presence of the free (non-adsorbed) MUS molecule. However, practically no acceleration was observed in either case (Fig. S17–S19, ESI†), providing conclusive evidence for the catalytic role of the MUS monolayer.

**Fig. 3:**
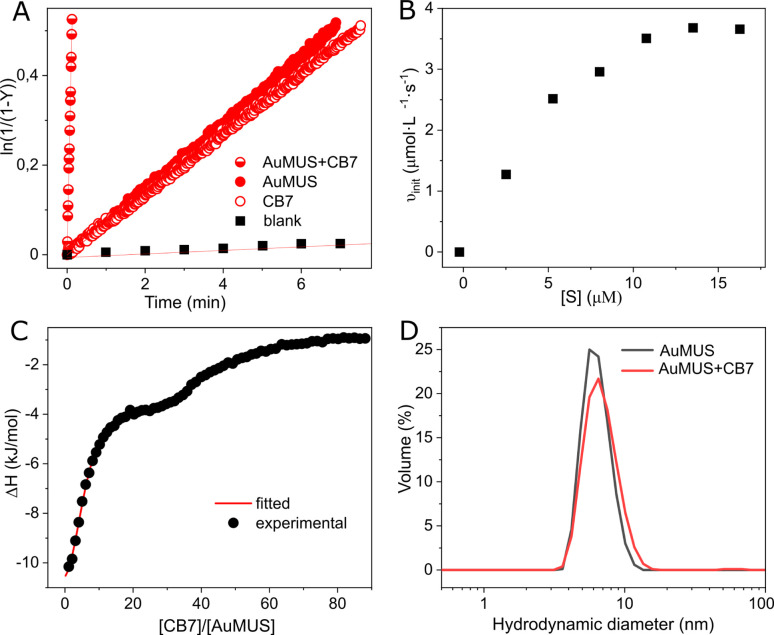
(A) Kinetic traces for the reaction progress in water (blank), in the presence of the macrocycle (1 equiv.), AuMUS (4 equiv.), and their mixture (1 : 4), [*S*] = 0.01 mM, water, pH = 6 (adjusted with HCl/NaOH), 298 K; (B) initial rates for CB7–AuMUS (1 : 4) at varying substrate concentrations; (C) titration of AuMUS with CB7, water, pH = 6 (adjusted with HCl/NaOH), 298 K; (D) the particle size distribution before (*d* = 6.41 ± 0.17 nm) and after the addition of CB7 (*d* = 6.86 ± 0.10 nm).

Subsequently, we introduced CB7 and AuMUS in varying ratios to the reaction mixture. We observed a significant increase in the reaction rate with the augmentation of both components. The most remarkable effect occurred at a 1 : 4 CB7/MUS molar ratio, which, given the presence of *ca.* 100 MUS on a single NP (Table S1, ESI†), corresponds to ≈4 mol% loading in terms of NPs, resulting in an acceleration factor of 1044, which is more than 50 times higher than that achieved by each component individually (Fig. 3A). Notably, the acceleration was consistently greater when an excess of immobilized MUS was present relative to CB7, indicating a limited number of adsorption (active) sites on the NP surface (Table 1). This limitation is further evidenced by the saturation kinetics observed upon the variation of the substrate concentration, a characteristic typical of enzymes and their mimics (Fig. 3B). Introducing a competitive inhibitor restricts access to these adsorption sites, resulting in a decrease in the reaction rate (Fig. S21, ESI†).

**Table 1:** Rate constants and acceleration factors defined as *α* = *k*_obs_/*k*_w_, where *k*_obs_ and *k*_w_ are reaction rates with and without CB7 and AuMUS at various ratios^a^

	CB7 : MUS ratio (in terms of MUS ligands)					
	1 : 4	4 : 1	1 : 1	0.25 : 1	1 : 0.25	0.25 : 0.25
*α*	1044	133	201	33	26	9

a In relation to the substrate.

To substantiate this assumption, we conducted binding studies between the NPs and CB7. ITC analysis disclosed a sequential process in macrocycle binding, comprising two distinct events (Fig. 3C, Table S3, ESI†). The first event, distinguished by very strong complexation (*K*_a_ = 2.07 ± 0.07 × 10^6^ M^−1^), corresponds to the adsorption of five CB7 macrocycles on a single nanoparticle. Subsequently, the second event, marked by much weaker binding (*K*_a_ = 7.19 ± 0.10 × 10^4^ M^−1^), involves forty-two interacting CB7 molecules.

**Fig. 4:**
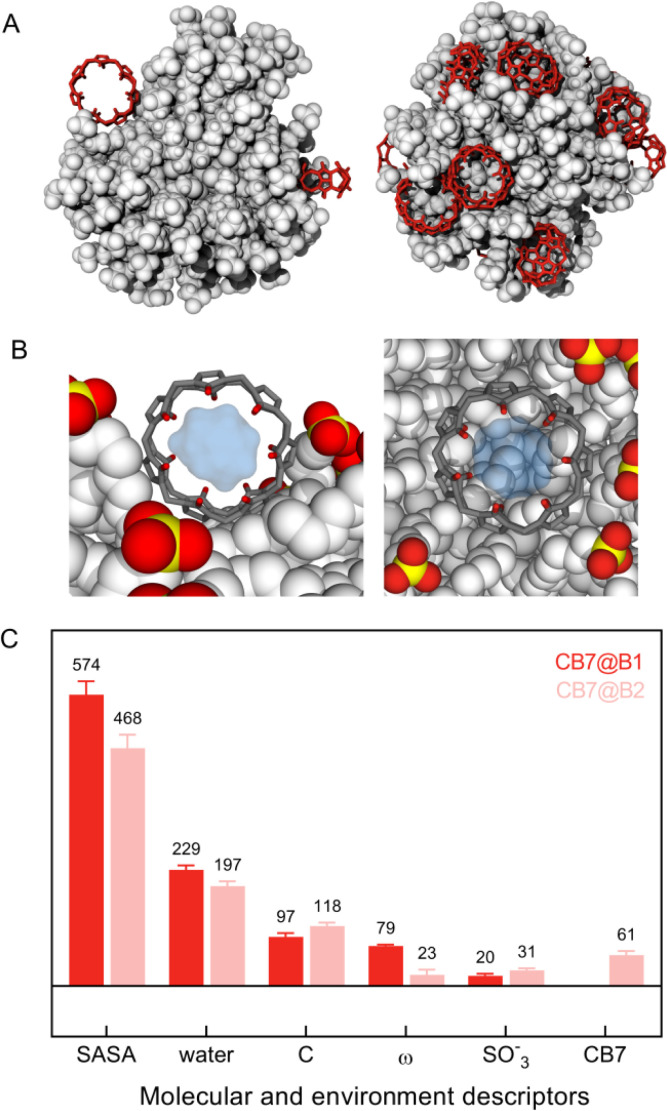
Location of CB7 molecules (red) at the onset (CB7@B1) (A, left) and at the late stage of binding (CB7@B2) (A, right) with AuMUS NPs (white). Structures are extracted from MD calculations at increasing CB7 content. CB7@B1 denotes binding occurrences prior to monolayer structure's reorganization, whereas CB7@B2 refers to those after; (B) zoom views of the AuMUS/CB7 complex highlighting the chemical space around the macrocyclic cavity in CB7@B1 (left) and CB7@B2 (right) modes; (C) comparison of molecular features and local environments pertaining to CB7@B1 and CB7@B2. Descriptors (from left to right): solvent accessible surface area (SASA, Å^2^), number of water molecules, number of C atoms belonging to MUS ligands, orientation of the macrocycle's central axis with respect to the direction connecting the center-of-mass of CB7 and NPs (*ω*, °), number of S/O atoms of SO_3_^−^ groups, and number of heavy atoms of another CB7 molecule. Environments are assigned for each CB7 accounting for all atoms within the first peak of the radial distribution function from the center of the macrocycle.

MD calculations unveiled that the first binding event (hereafter named CB7@B1) matches with the spontaneous attachment of the macrocycles onto the NP surface in the space left free by clustering of MUS ligands in bundles (Fig. 4A, left panel and Fig. S24†). The rings are thus accommodated within the internal voids facilitating contact with neighbouring chains. This arrangement enables the surrounding ligands to envelop the macrocycle and concurrently maintains an open pathway to the hydrophobic cavity. Accordingly, CB7 molecules initially bind to the NP by leveraging the bundle-void pattern.

Beyond a few CB7 units, the subsequent incorporation of CB7 molecules disrupts the clustering of ligands entirely, compelling the monolayer to undergo structural reconstruction (Fig. S25†). As a consequence of that, a second binding mode (named CB7@B2) arises, where CB7 has now a different orientation within the monolayer (Fig. 4A, right panel, and Fig. S26†). This is accompanied by a substantial decrease in the associated free energy of binding, namely −6.4 kcal mol^−1^ against −10.6 kcal mol^−1^ found for CB7@B1, which is consistent with weaker affinity found in ITC measurements for second binding events. The embedment of CB7 within the monolayer is supported by DLS studies, which showed only a slight decrease in the hydrodynamic diameter of AuNPs after the addition of the macrocycle (Fig. 3D). The binding of CB7 with AuMUS is likely primarily driven by the interactions between the electropositive outer macrocycle surface and the surrounding anionic sulfonate groups in the organic monolayer, involving (i) hydrogen bonding between CH or CH_2_ groups on the external walls of the macrocycle and SO_3_ groups of MUS ligands and (ii) ion–dipole interactions between the carbonyl carbon atoms of CB7 and the sulfonate groups of MUS ligands.^38^

We then compared the local environment around CB7@B1 and CB7@B2, as signatures of the binding settings, by monitoring key environment descriptors, along with molecular features of CB7 (Fig. 4C). A major difference is the reduced accessibility to the macrocycle for CB7@B2 due to the increased embedding in the monolayer manifested by the decreased solvent accessible surface area (SASA) and angle between the central axes of the macrocycle and nanoparticle (*ω*). The inner cavity of CB7 still remains accessible; however, the more hidden position enables incoming (or leaving) molecules entering (or exiting) it from one side only, unlike CB7@B1, which allowed access from two sides. Note that there is no significant variation in the available cavity volume for CB7 in solution and on the surface (Fig. 4B and S27†).

We therefore posit that CB7@B1, *i.e.*, only a handful macrocyclic molecules among a multitude, is likely to be responsible for the catalytic performance of suprazymes, allowing for the full entry of the substrate into the macrocyclic cavity and collective electrostatic interactions of charged intermediates with both carbonyl portals and MUS ligands within the hydrophobic environment spanning from the inner cavity of CB7 to the monolayer's interior. This conjecture gains support from the heightened reaction rate of the oxime formation in acetonitrile (*α* = 11, Fig. S20, ESI†), a less polar medium compared to water. Concurrently, other effects, such as the improved pre-organization of substrates or the proton donor/acceptor abilities of the encapsulated water,^39^ may also be at play. Hence, unravelling the mechanism of catalysis requires further in-depth computational efforts, which are presently underway in our group.

## Conclusions

In summary, we have demonstrated that gold nanoparticles coated with anionic ligands decrease the local pH on their surfaces, promoting the acid-catalyzed oximation reaction. Moreover, when combined with macrocyclic acid substitutes such as cucurbiturils, their catalytic efficacy increases dramatically, resulting in a reaction acceleration of three orders of magnitude. The observed behavior is due to the emergence of a suprastructure, referred to as “suprazyme”, specifically composed of macrocycle molecules nested within the organic monolayer on the nanoparticle surface. Despite the elusive nature of this new species compared to traditional enzyme mimics, which typically have monodisperse and well-defined active sites, this work paves the way for enhancing the performance of molecular catalysts through non-covalent embedding into organic monolayers and the modulation of their composition and structure.

## Data availability

Raw data for this article are available at RepOD at https://doi.org/10.18150/DTSHJ5. Processed data supporting this article have been included as part of the ESI.†

## Author contributions

Conceptualization, V. S.; data curation, P. H. and M. F.; formal analysis, P. P. and V. S.; funding acquisition, P. P. and V. S.; investigation, P. H. and M. F.; methodology, P. P. and V. S.; supervision, P. P. and V. S.; writing – original draft, P. P. and V. S.; writing – review & editing, P. H., M. F., P. P. and V. S.

## Conflicts of interest

There are no conflicts to declare.

## Supplementary Material

SC-OLF-D4SC04577G-s001
