# Supplementary material for: Creating a suprazyme: integrating a molecular enzyme mimic with a nanozyme for enhanced catalysis
Source: Chem Sci. 2024 Sep 26;15(40):16480–4. doi: 10.1039/d4sc04577g (PMC11450938; doi:10.1039/d4sc04577g)
Supplement: SC-015-D4SC04577G-s001 [file SC-015-D4SC04577G-s001.pdf]

Supplementary Information (SI) for Chemical Science.
This journal is © The Royal Society of Chemistry 2024

Supplementary Information

Creating a suprazyme: integrating a molecular enzyme mimic with a nanozyme for enhanced catalysis

Pavlo Hyziuk,[a] Matteo Flaibani,[b] Paola Posocco,*[b] and Volodymyr Sashuk*[a]

[a] Institute of Physical Chemistry, Polish Academy of Sciences, Kasprzaka 44/52, 01-224 Warsaw, Poland

[b] Department of Engineering and Architecture, University of Trieste, 34127 Trieste, Italy

E-mail: paola.posocco@dia.units.it, vsashuk@ichf.edu.pl

Table of Contents

1. General Information S1
  1.1 Experimental methods S1
  1.2 Computational methods S2
2. Synthesis of aldehyde and oxime S5
3. Synthesis and functionalization of AuNPs S10
4. Characterization of AuNPs S11
5. pKa measurements S14
6. Binding studies S16
7. Kinetic studies S17
8. Computational results S22
9. Appendix S24
  9.1 MD input files S24
  9.2 MD molecular starting structures S26
10. References S33

1. General Information

1.1 Experimental methods

All chemicals were procured as reagent-grade materials from commercial suppliers, including Merck, TCI, and Thermo Fisher Scientific, and were utilized without additional purification. The solvents employed, sourced from Merck, ChemPur, and PoCh, met analytical-grade standards. Deuterated solvents were acquired from Armar Chemicals. Experiments were conducted at room temperature unless otherwise specified. NMR spectra were recorded on a Bruker 400 MHz instrument and analyzed using MestReNova software. Chemical shifts (δ) are reported in ppm relative to TMS, and coupling constants (J) are expressed in Hz.

Quartz cuvettes were obtained from Hellma Analytics. Absorbance spectra were recorded using the Evolution220 spectrophotometer from Thermo Fisher Scientific and analyzed with Origin software. Automatic mixing in the cuvette was facilitated using Peltier thermostating accessories from Thermo Fisher Scientific. High-resolution ESI mass spectra were recorded on a SYNAPT spectrometer, and TEM images were captured on an FEI TECNAI instrument, with analysis conducted using the ImageJ program. DLS data were obtained from Malvern Zetasizer. Thermogravimetric analysis (TGA) was performed using the Mettler Toledo TGA/DSC 3+ instrument. pH measurements were conducted using the HI 3220 pH Meter equipped with an InLab® Micro glass electrode from Mettler Toledo. Isothermal titration experiments were carried out on a Malvern MicroCal PEAQ-ITC at 25°C in deionized water obtained from a Milli-Q station with a resistivity of 18.3 MΩ·cm.

1.2 Computational methods

Atomistic models

An icosahedron of gold (Au) atoms with a diameter of 2.5 nm was used as core of the nanoparticle (NP) model, with a material density of 19.3 g/cm^3 and a lattice constant of ~ 0.41 nm, for a total of ~ 309 gold atoms. A uniform distribution of MUS ligands was placed on the NP surface, using an amount of molecules matching the experimental chain density value (see section S4). The model was built using the Atomistic Simulation Environment (ASE)[1] set of tools and the mBuild[2] software. The parameters of the INTERFACE[3] force field for metals were used to describe the interactions between Au atoms, while the MUS ligand was parametrized according to the gaff2[4] force field. The parmchk2 software, available within the AMBER 22 suite of programs, was employed to test if all the parameters required for the MUS ligand were already available in the gaff2 force field. No missing parameters required an external derivation. The atomic partial charges of the molecule derived using the RED server, applying the RESP fitting method.[5-9] CB7 and aldehyde molecules were parametrized following the same procedure described for MUS ligands, with all parameters being already available in the gaff2 force field. A cubic box of water molecules (TIP3P) was placed around the system of interest using the *tleap* software of the AMBER 22[10] suite of programs, with at least 18 Å of solvent from each solute atom (average number of water molecules equal to ~ 40000). Counterions were introduced when it was necessary to neutralize the system.

Molecular dynamics (MD) simulations of the AuMUS

The AuMUS was simulated using the AMBER 22 suite of programs, exploiting the acceleration provided by the GPU (CUDA) version of the PMEMD engine[11-13] and replicated for three times. The solvated system was minimized in two steps, keeping all the solute atoms (nanoparticle and counterions) fixed in the first part of the procedure, allowing for the relaxation of the solvent molecules. A value of 10 Å was used as cutoff for the non-bonded interactions. The system was subsequently brought to the final simulation temperature of 300 K, slowly increasing the value in a 100 ps simulation performed in the canonical NVT ensemble (integration step of 1 fs). An equilibration stage of 10 ns at constant temperature and pressure (300 K and 1 atm) was performed using first the Berendsen barostat and then switching to the Monte Carlo barostat for 10 ns (integration step of 2 fs). The final production phase was performed in the NPT ensemble for 100 ns, employing the Monte Carlo barostat and applying the SHAKE algorithm to restrain the bonds involving hydrogen atoms. Periodic Boundary Conditions (PBC) were employed, computing the electrostatic interactions with the Particle Mesh

Ewald (PME) algorithm, considering the interactions below a cutoff of 10 Å. A restraint on the positions of the Au atoms and sulfurs of the thiol groups of the ligands was applied during the simulations, with a force constant of 200 kcal/mol ·$Å^2$. A restraint on the gold atoms was applied in all simulations involving the AuMUS nanoparticle.

MD simulation of the CB7

CB7 was simulated using the PMEMD engine, with PBC conditions and employing the PME algorithm to treat the electrostatic interactions, with a cutoff of 10 Å. The same cutoff was used for the non-bonded interactions. The crystallographic structure of CB7 was employed as the starting model, downloaded from the free available Protein Data Bank (PDB) https://www.rcsb.org/ (PDB ID QQ7). The system was initially solvated with water molecules and then minimized in two stages: first, with the CB7 kept fixed, followed by a full minimization of the entire system. A heating step in the NVT ensemble was performed after the minimization, bringing the system to the final temperature of 300 K, in a simulation time of 100 ps (integration step of 1 fs). Before the final production phase, the system was equilibrated for 20 ns (integration step of 2 fs) at a constant temperature of 300 K and constant pressure of 1 atm, employing the Berendsen barostat for the first 10 ns and the Monte Carlo barostat for the last 10 ns of equilibration. The production phase was performed in the NPT ensemble for 100 ns, keeping the pressure constant with the Monte Carlo barostat. The bonds involving hydrogen atoms were restrained using the SHAKE algorithm. Three replicas were carried out.

MD simulation of the aldehyde

The PMEMD engine was employed to simulate the aldehyde with PBC conditions, handling the electrostatic and non-bonded interactions as described in the two previous paragraphs. Bonds involving hydrogen atoms were restrained with the SHAKE algorithm. The starting structure of the aldehyde molecule was created with a molecule editor. The system was solvated and neutralized with two counterions, followed by the minimization procedure described above, keeping both the aldehyde and counterions fixed in the first step. The final temperature of 300 K was reached with a 100 ps simulation (integration step of 1 fs) in the NVT ensemble, followed by an equilibration step in the NPT ensemble (integration step of 2 fs) before starting the final production of 100 ns. Three replicas were considered.

CB7-aldehyde interaction

The study of the host-guest complex was performed starting from the two equilibrated molecules, obtained from the MD simulations described above, and replicated three times. The substrate was manually inserted into the macrocycle, placing the aromatic ring inside the cavity. This choice was motivated by the hydrophobic nature of both the aromatic moiety and the CB7 cavity, allowing also for charge-dipole interaction between a charged group of the aldehyde with one carbonyl rim of the CB7. The complex was then solvated, neutralized with the addition of counterions, followed by the two-step minimization procedure reported in the previous paragraphs. The system was brought at the final temperature of 300 K in the NVT ensemble and subsequently equilibrated at constant temperature and pressure before the final production phase. The complex was simulated for 100 ns in the NPT ensemble, monitoring the aldehyde dynamics inside the macrocycle.

AuMUS-CB7 interaction

The study of the interaction between the NP and CB7 was performed starting from their equilibrated molecular models, extrapolated from the MD simulations described above, and performed three times using three different initial structures. A single CB7 molecule was manually placed at a distance higher than the cutoff used for the non-bonded interactions, to avoid effects that could arise from the initial configuration. The system was then solvated and counterions were added to reach neutralization. After being minimized and equilibrated at the final temperature and pressure, the system underwent a simulation of 100 ns in the NPT ensemble before the subsequent addition of the next CB7 molecule; this procedure aimed to mimic a sequential binding process somehow similar to that occurring during ITC experiments. The system was again equilibrated and simulated in the NPT ensemble each time a macrocycle was added, allowing the system to relax. Simulations proceeded in this way until a total of 12 CB7 units was bound to the NP; this was the threshold beyond which any further addition would have required more user intervention, potentially rendering the resulting simulations susceptible to artifacts.

AuMUS-aldehyde interaction

The study of the interaction between the NP and substrate was conducted employing their equilibrated structures, obtained from the MD simulations described above, and repeated three times. Aldehyde molecules were placed in a spherical layer around the NP using the PACKMOL software[14] in a number that corresponds to the experimental binding studies (see section S6). Preliminary MD runs and observables from ITC experiments suggested us that a computationally time-consuming stepwise approach to study AuMUS-aldehyde complexation was unnecessary.[15] After the addition of the aldehyde molecules the system was solvated and neutralized, with counterions to balance the negative and positive charge of ligands and aldehydes. The system was subsequently minimized and equilibrated at the final temperature and pressure before being simulated for 100 ns in the NPT ensemble, monitoring the behavior of the system.

CB7-aldehyde umbrella sampling calculations

The binding free energy relative to the substrate-CB7 complexation was retrieved by means of umbrella sampling (US) calculations, extracting the substrate from the cavity of the CB7. The starting point was the equilibrated structure of the substrate-CB7 complex, to which a set of dummy atoms was added. These dummy atoms, whose positions were restrained during simulations with a force constant of 100 kcal/mol $\cdot$Å^2, were employed as anchor points for sampling, allowing us to pull the aldehyde molecule out of the macrocycle along a straight line, thus studying the direct association process.[16] The distance between one dummy atom and the carbon atom of the carbonylic group of the aldehyde was chosen as single collective variable to monitor. A total of 95 windows were simulated, spanning from 2.85 to 49.85 Å, spaced by 0.50 Å. A constant of 10 kcal/mol $\cdot$Å^2 was used to define the harmonic potential. Every single window was simulated for a total of 1.5 ns, discarding the first 500 ps as equilibration. The simulations were performed in series, starting the simulation of each window from the last frame of the previous one. We employed the same parameters used for the MD simulations. The binding free energy was obtained from the free energy profile reconstructed using the Weighted Histogram Analysis Method (WHAM)[17-20] on three replica. The effect of the orientational restraint was accounted calculating the corresponding correction terms, as reported in literature.[21,22]

AuMUS-CB7 umbrella sampling calculations

The free energy relative to binding of the macrocycle to the NP was calculated by means US calculations, pulling the CB7 away from the monolayer. As starting point, we selected CB7@B1s and CB7@B2s as detected from the unbiased MD calculations. Three replicas for each binding mode were averaged. The distance between the center of mass of the macrocycle and the nearest sulfur atom bound to the gold surface was chosen as single collective variable. The macrocycles were pulled away from the NP along a straight line, restraining the angle between the center of mass (COM) of the CB7, the chosen sulfur atom and the center of the NP, until a distance of 30 Å was reached. Approximatively 45 windows, spaced by 0.5 Å, were sampled for each CB7, depending on the starting distance. A constant of 25 kcal/mol ·Å^2 was employed for the harmonic potential restraining the distance, while a constant of 1000 kcal/mol ·rad^2 was used to keep the angle fixed during the pulling. Every single window was simulated for a total of 2.5 ns, discarding the first 500 ps as equilibration.

2. Synthesis of aldehyde and oxime

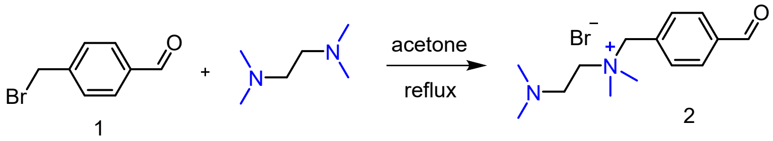

2-(Dimethylamino)-N-(4-formylbenzyl)-N,N-dimethylethan-1-aminium bromide (2): Compound **1**[23] (109 mg, 0.548 mmol) was dissolved in 2 mL of acetone. To this solution, 1,4-tetramethylenediamine (1.64 mL, 10.96 mmol) was added with stirring at room temperature. The reaction mixture was then refluxed overnight. Afterward, the solvent was concentrated under reduced pressure, yielding a pure off-white solid (163.1 mg, 94%) that was utilized in the subsequent stage. ^{1}H NMR (400 MHz, acetonitrile-*d$_3$*): δ 10.09 (s, 1H), 8.05 – 7.99 (d, J = 8.2 Hz, 2H), 7.78 – 7.72 (d, J = 8.2 Hz, 2H), 4.65 (s, 2H), 3.47 – 3.41 (t, J = 6.0 Hz, 2H), 3.05 (s, 6H), 2.79 – 2.71 (t, J = 6.0 Hz, 2H), 2.25 (s, 6H). ^{13}C NMR (100 MHz, acetonitrile-*d$_3$*): δ 192.41, 137.67, 134.11, 134.01, 129.7, 66.98, 61.35, 53.02, 49.84, 44.6. HRMS (ESI) m/z: calc. for $C_{14}H_{23}N_2O$: 235.1810 [M]$^+$; found: 235.1814.

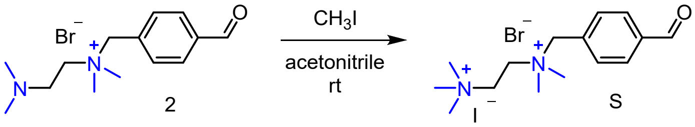

N^1-(4-formylbenzyl)-N^1,N^1,N^2,N^2,N^2-pentamethylethane-1,2-diaminium bromide iodide (S): Compound **2** (163.1 mg, 0.517 mmol) was dissolved in 3.5 mL of acetonitrile. To this solution, iodomethane (0.322 mL, 5.17 mmol) was added with stirring at room temperature. The reaction mixture was stirred overnight at room temperature. Afterward, the solvent was concentrated under reduced pressure. The resulting solid residue was redissolved in acetonitrile, and diethyl ether was introduced. The precipitate was collected by centrifugation, washed with diethyl ether (3x10 mL), and dried under vacuum to afford the target aldehyde **S** as an off-white powder (214 mg, 91% yield).^{1}H NMR (400 MHz, acetonitrile-*d$_3$*): δ 10.11 (s, 1H), 8.08 – 8.02 (d, J = 8.2 Hz, 2H), 7.87 (d, J = 8.2 Hz, 2H), 4.86 (s, 2H), 4.12 (s, 4H), 3.27 (s, 9H), 3.16 (s, 6H). ^{13}C NMR (100 MHz, acetonitrile-*d$_3$*): δ 193.33, 139.06, 135.21, 133.59, 130.94, 68.55, 58.71, 57.60, 55.11, 51.38.

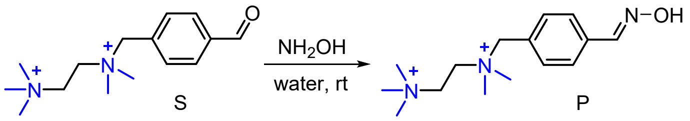

N[1]-(4-((hydroxyimino)methyl)benzyl)-N[1],N[1],N[2],N[2],N[2]-pentamethylethane-1,2-diaminium bromide iodide (P): The experiment was similar to UV-Vis studies (pH=6, 298 K) but conducted in deuterated water at increased concentrations of the substrate (2mM) and hydroxylamine (40 mM) to enable NMR measurements. ^{1}H NMR (400 MHz, acetonitrile-d_3): δ 8.19 (s, 1H), 7.74 (d, J = 8.1 Hz, 2H), 7.66 (d, J = 8.1 Hz, 2H), 4.74 (s, 2H), 4.15 – 3.99 (m, 4H), 3.26 (s, 9H), 3.13 (s, 6H). ^{13}C NMR (100 MHz, acetonitrile-d_3): δ 149.27, 136.61, 134.71, 128.60, 128.31, 69.34, 58.72, 57.21, 55.10, 51.21.

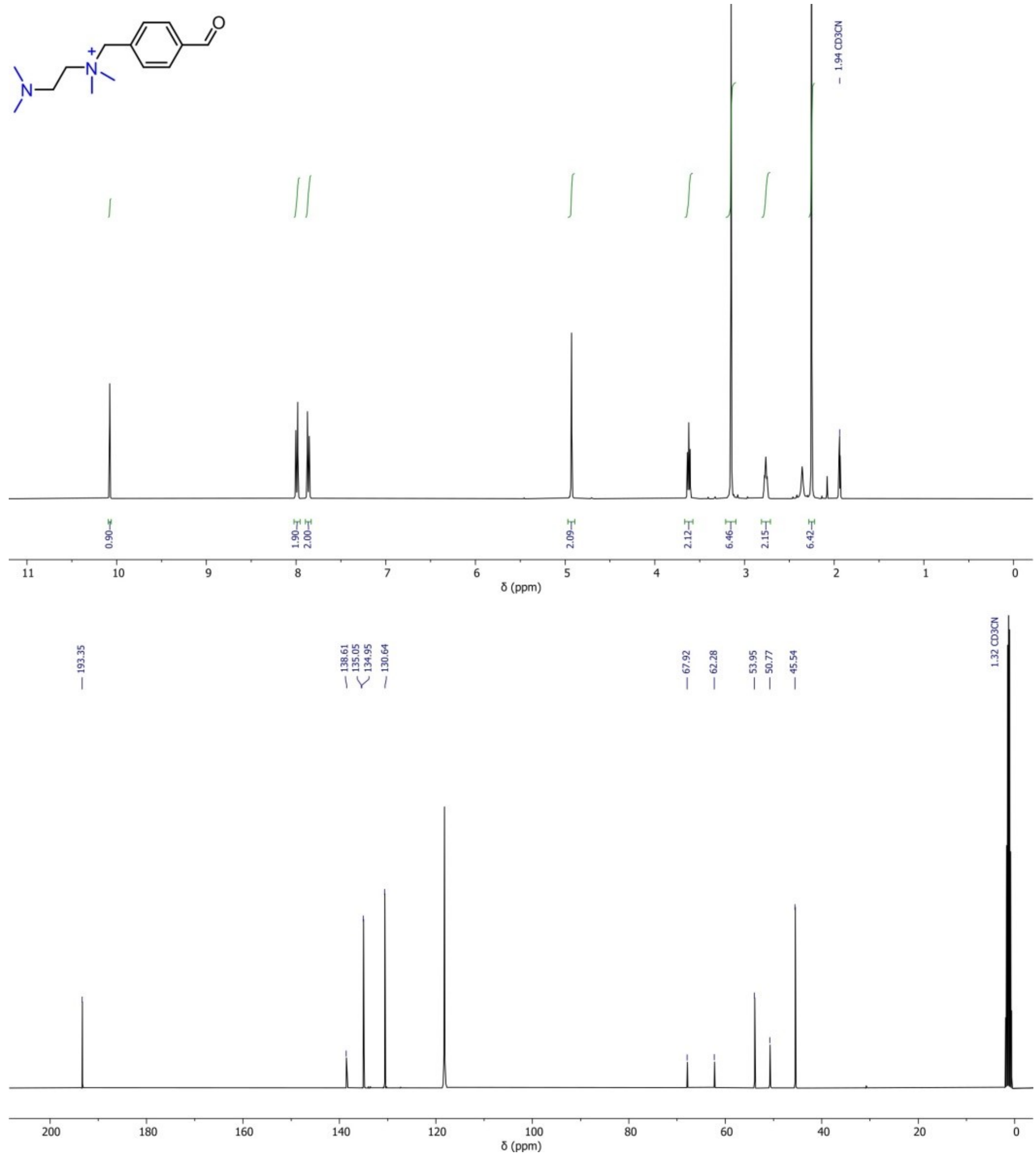

Fig. S1 ^{1}H and ^{13}C NMR spectra of **2** in CD_3CN at 298 K.

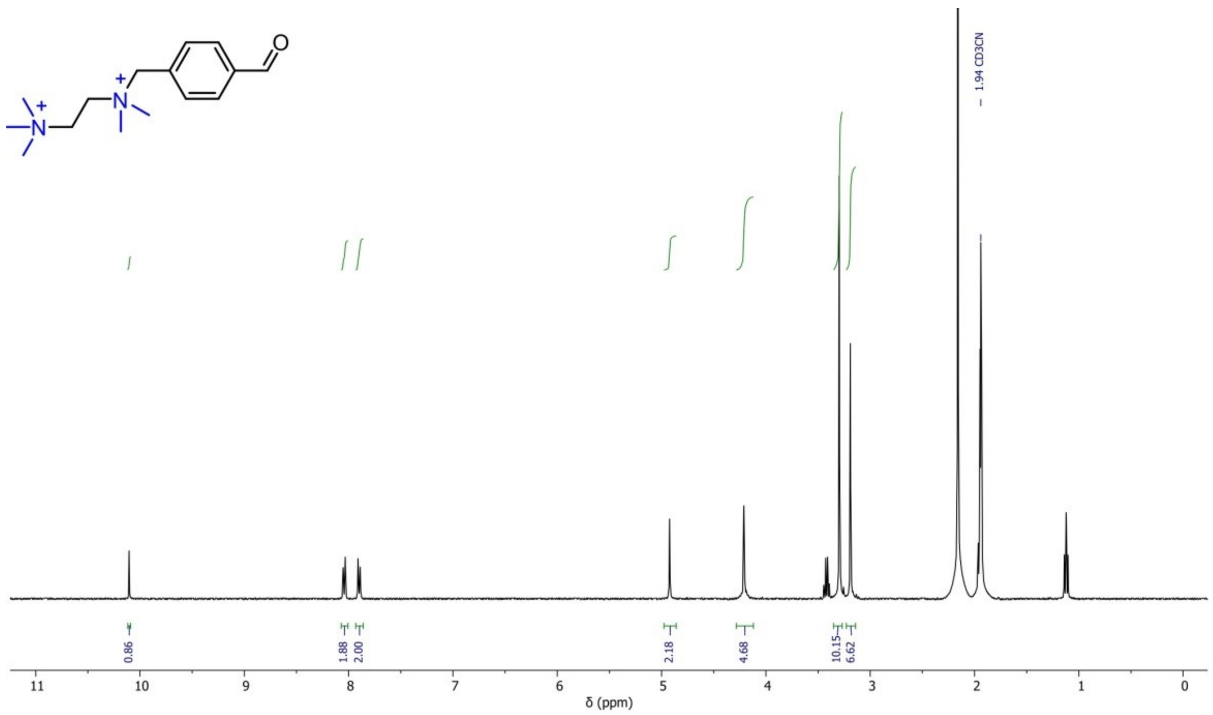

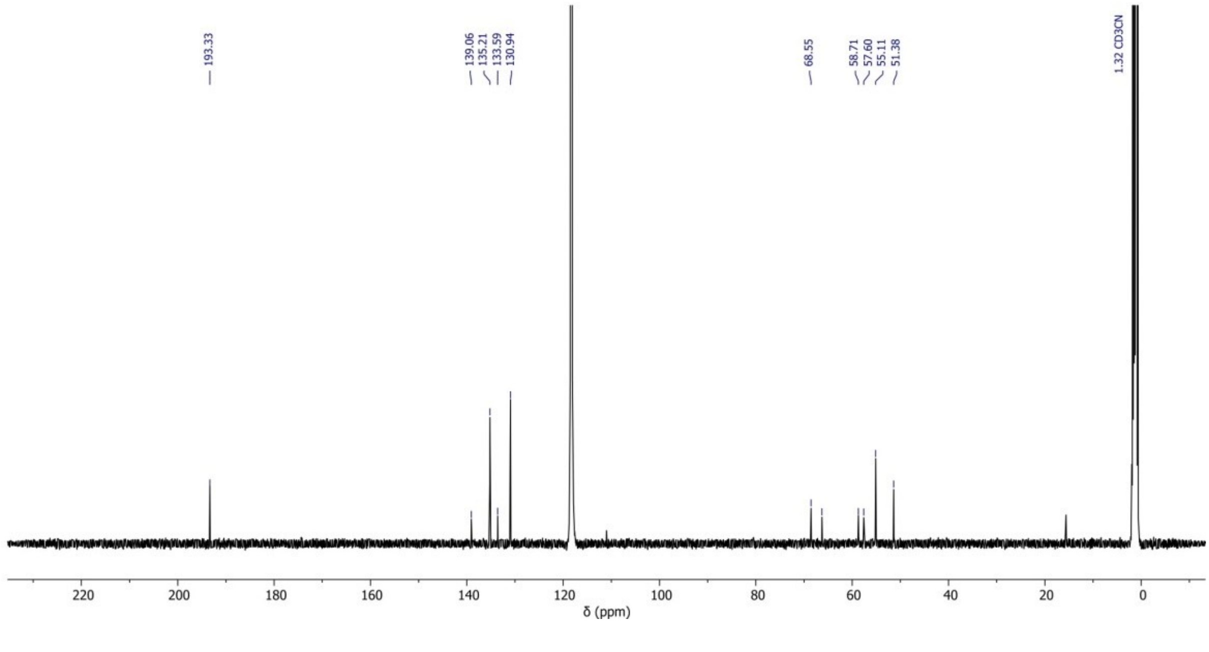

Fig. S2 ^{1}H and ^{13}C NMR spectra of **S** in CD_3CN at 298 K.

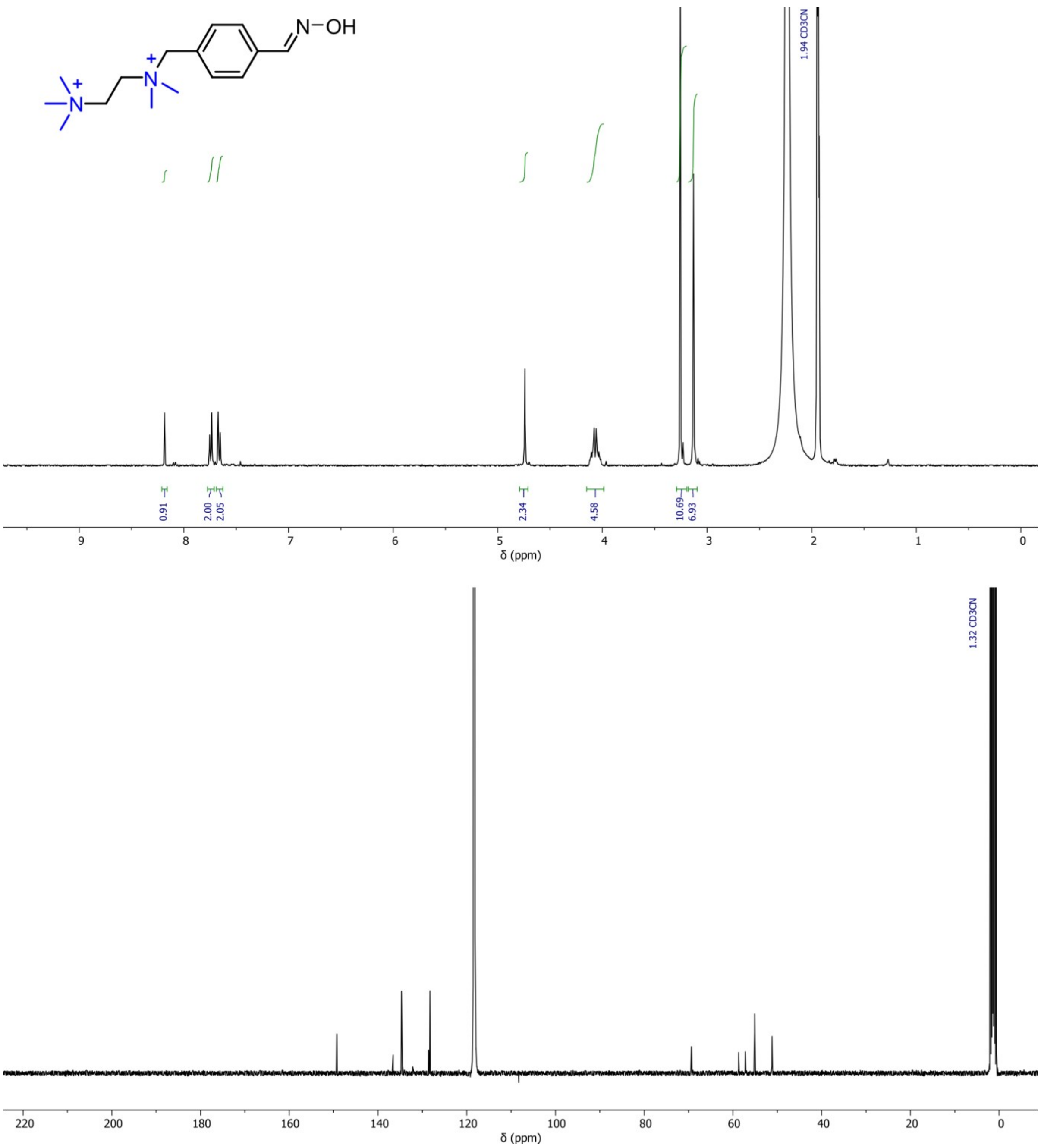

Fig. S3 ^{1}H and ^{13}C NMR spectra of **P** in CD_3CN at 298 K.

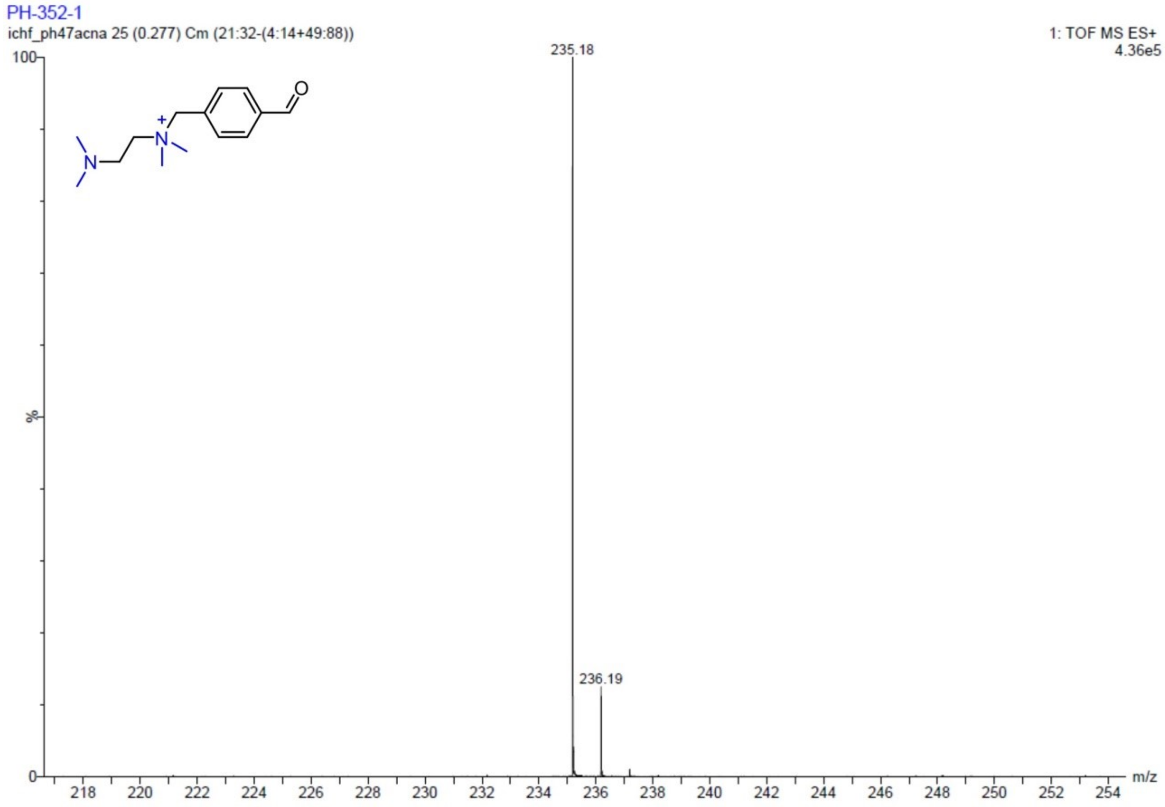

Fig. S4 Mass spectrum of **2** under electrospray ionization in positive ion mode.

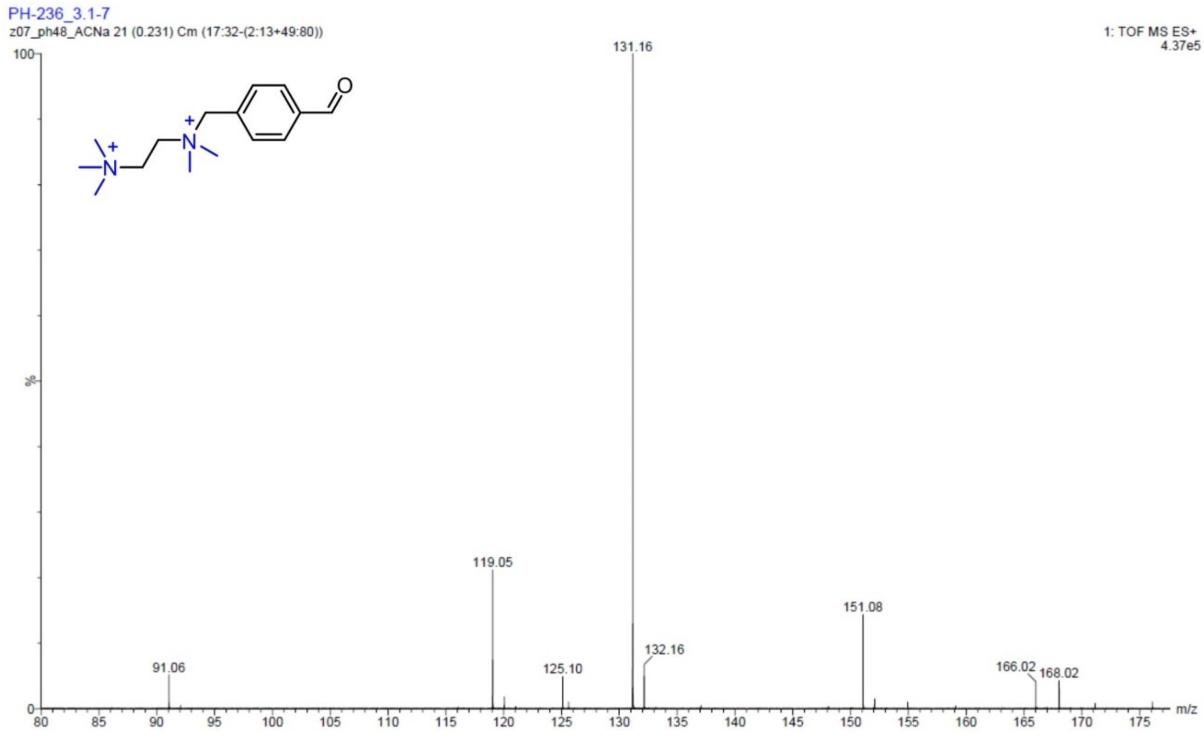

Fig. S5 Mass spectrum of **S** under electrospray ionization in positive ion mode.

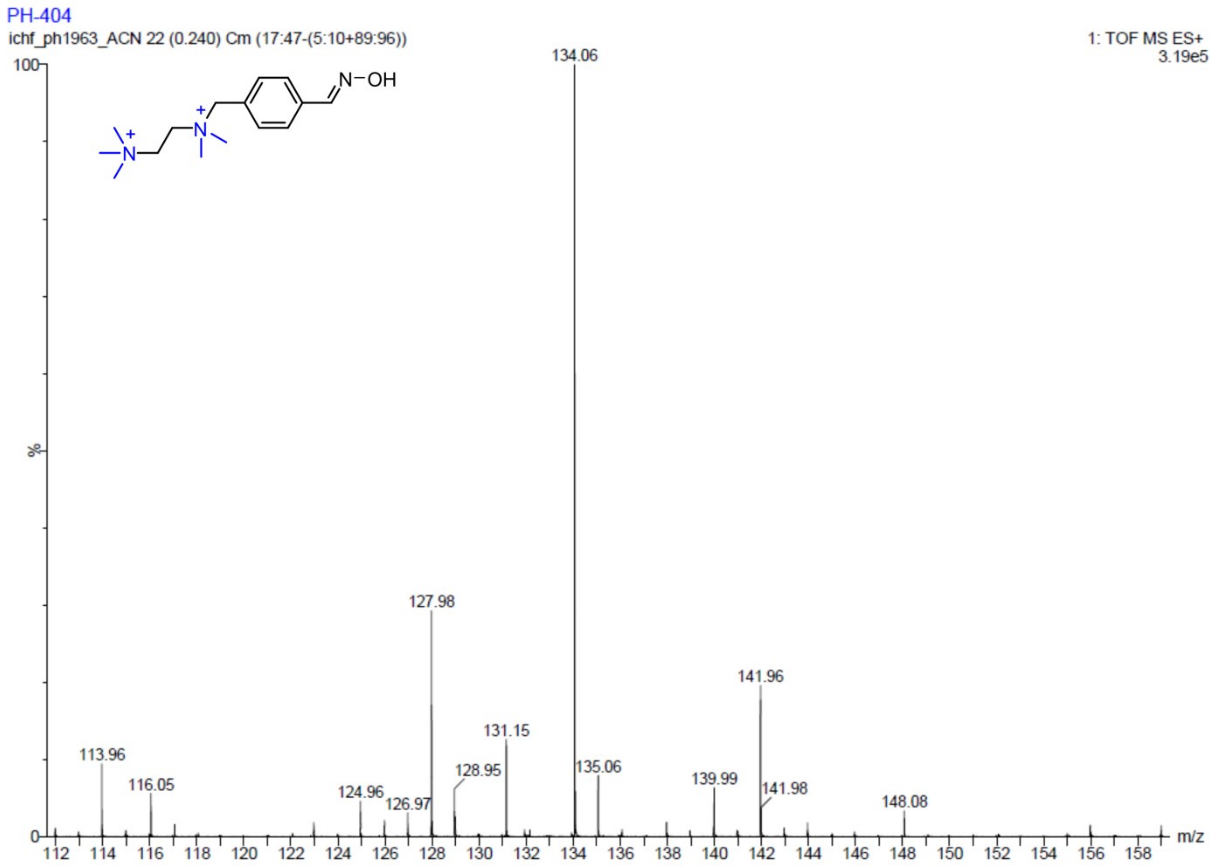

Fig. S6 Mass spectrum of **P** under electrospray ionization in positive ion mode.

3. Synthesis and functionalization of AuNPs

Synthesis of thiol-coated AuNPs (AuMUS, AuTMA, AuPEG)

$HAuCl_4 \cdot 3H_2O$ (9.75 mg, 0.025 mmol) together with dodecylamine (90 mg, 0.486 mmol) were dissolved in 2.5 mL of 0.1M didodecyldimethylammonium bromide (DDAB) toluene solution, and reduced by adding tetrabutylammonium borohydride (25 mg dissolved in 1 ml of 0.1M DDAB toluene solution) in a single portion under vigorous stirring. The resulting mixture was stirred for 1.5 hours. Subsequently, 10.5 mL of methanol was added, and precipitated gold nanoparticles (AuNPs) were collected by centrifugation at 1000 RPM.

The AuNPs dissolved in chloroform (3.5 mL) were added dropwise to a solution of the ligand, either MUS[24] (3.43 mg, 0.012 mmol) or TMA[25] (1.86 mg, 0.007 mmol), in methanol (7 mL) with vigorous stirring. In the case of the PEG[26] ligand (5.26 mg, 0.025 mmol), the procedure was similar, except chloroform (2 mL) was used instead of methanol. The resulting mixture was stirred for an additional 1.5 hours.

Purification of AuMUS

AuMUS NPs were collected by centrifugation and washed with methanol (3x5 mL). Finally, the NP sediment was dried and then redispersed in 951 µL of deionized water or heavy water, resulting in a 21.44 mM (in terms of gold atoms) NP dispersion.

Purification of AuPEG

AuTMA NPs were precipitated by pouring the reaction mixture into pentane (7 ml) and collected by centrifugation. The NP sediment was redispersed in CH_3OH (100 µL), precipitated by pentane (7 mL) and centrifuged. The procedure was repeated two times. Finally, the NPs were dried and redispersed in 1.371 mL of deionized water to give 15.32 mM (in terms of gold) NP dispersion.

Purification of AuPEG

AuPEG NPs were precipitated by pouring the reaction mixture into pentane (15 ml) and collected by centrifugation. The NP sediment was redispersed in $CHCl_3$ (1 mL), precipitated by pentane (15 mL) and centrifuged. The procedure was repeated two times. Finally, the NPs were dried and redispersed in 1.233 mL of deionized water to give 15.68 mM (in terms of gold) NP dispersion.

4. Characterization of AuNPs

Sample preparation for TEM

AuMUS dispersions were dropcast onto 300 mesh Cu grids and analyzed using TEM.

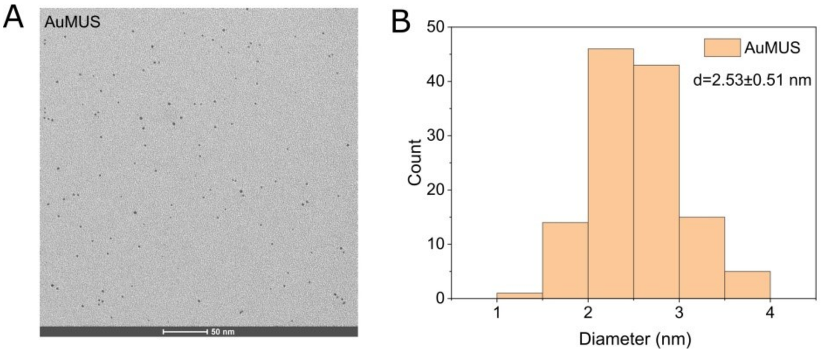

Fig. S7 TEM micrograph and size analysis of AuMUS.

Sample preparation for TGA

AuMUS, AuTMA and AuPEG dispersions were transferred to ceramic crucibles, dried and subjected to TGA.

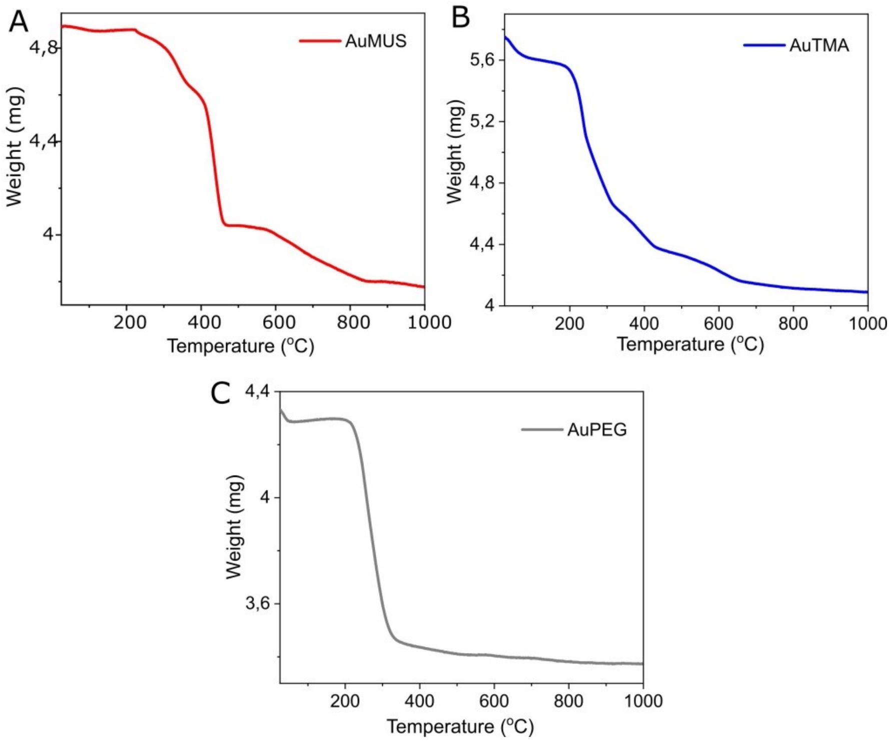

Fig. S8 Thermograms of AuNPs coated with MUS (A), TMA (B) and PEG (C) monolayers.

Ligand shell quantification

The ligand density (ρ_{lig}) can be calculated by dividing the total number of ligands (N_{lig}) by the total surface area of gold nanoparticles (S_{total}) using the formula:

$$\rho_{lig} = \frac{N_{lig}}{S_{total}}$$

The total number of ligands (N_{lig}) can be determined from:

$$N_{lig} = \frac{m_{lig}}{M_{lig}} \cdot N_a,$$

where m_{lig} is the total mass of adsorbed ligands (equal to the total weight loss during TGA), M_{lig} is the molecular weight of the ligand, and N_a is Avogadro's number.

The total area of gold nanoparticles (S_{total}) can be estimated from:

$S_{total} = S_{AuNP} N_{total}$,

where S_{AuNP} is the surface area of a single nanoparticle, given by:

$$S_{AuNP} = 4\pi r_{AuNP}^{\ 2}$$

and N_{total} is the total number of nanoparticles calculated using the formula for the volume of a sphere:

$$V_{AuNP} = \frac{4}{3}\pi r_{AuNP}^{\ 3},$$

where r_{AuNP} ($\approx$ 1.27 nm) is the average radius of the gold core

the mass of a single nanoparticle (m_{AuNP}):

$$m_{AuNP} = \rho_{Au} V_{AuNP} = \rho \frac{4}{3}\pi r_{AuNP}^{\ 3},$$

where $\rho_{Au} = 19.3\ g/cm^3$ is the density of gold

and the weight of the sample after TGA (m_{total}):

$$N_{total} = \frac{m_{total}}{m_{AuNP}} = \frac{3m_{total}}{4\rho_{Au}\pi r_{AuNP}^{\ 3}}$$

To determine the number of ligands per single nanoparticle, the total number of ligands (N_{lig}) was divided by the total number of gold nanoparticles (N_{total}):

$$N_{lig}(per\ single\ AuNP) = \frac{N_{lig}}{N_{total}}$$

Table S1. Calculated ligand densities and quantities.

Sample	ρ_{lig}, $molecules/nm^2$	N_{lig} $(per\ single\ AuNP)$
AuMUS	4.7	96
AuTMA	6.6	133
AuPEG	6.4	129

5. pKa measurements

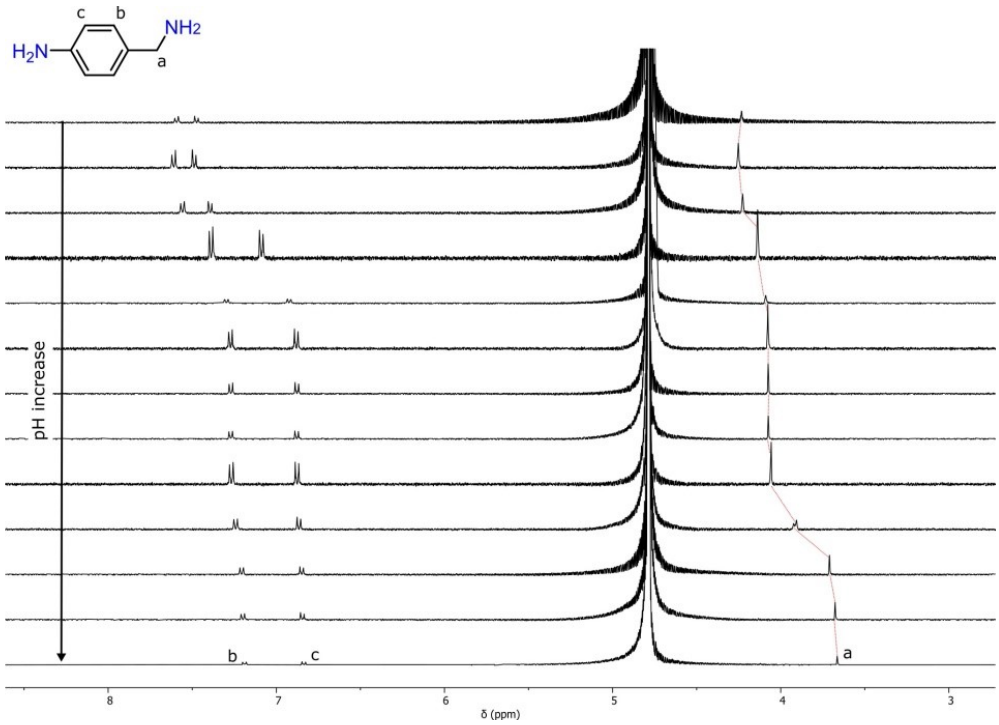

Fig. S9 ^1H NMR spectra of 4-aminobenzylamine at various pH_{obs} (D_2O, 298 K).

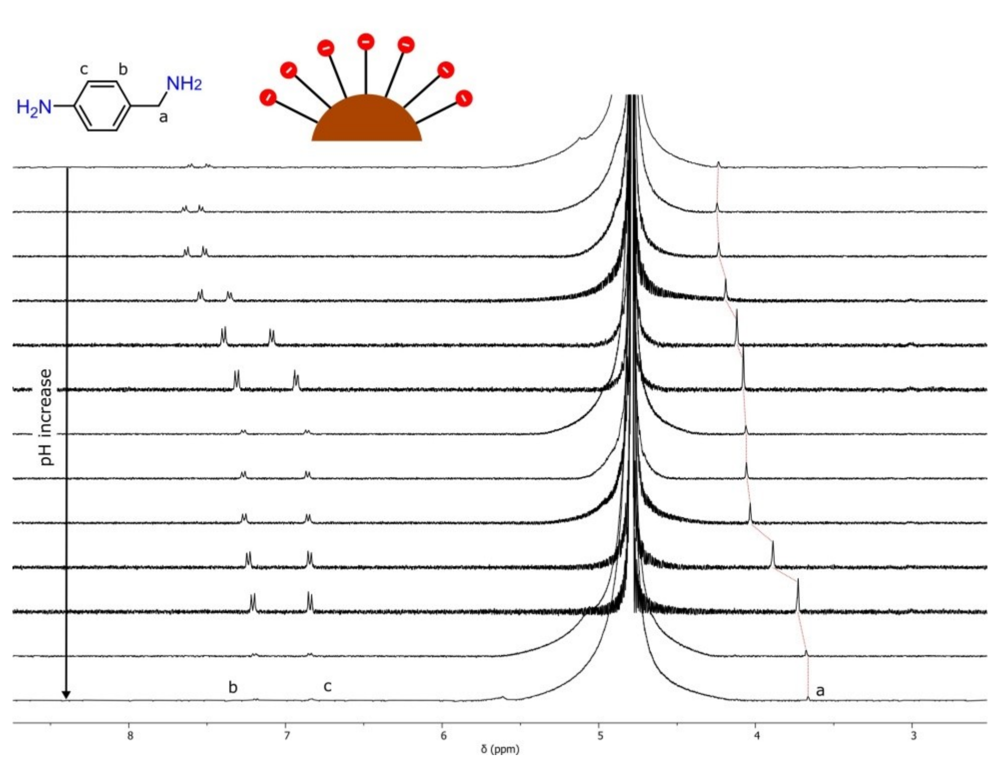

Fig. S10 ^{1}H NMR spectra of 4-aminobenzylamine in the presence of AuMUS (4 equiv) at various pH_{obs} (D_2O, 298 K).

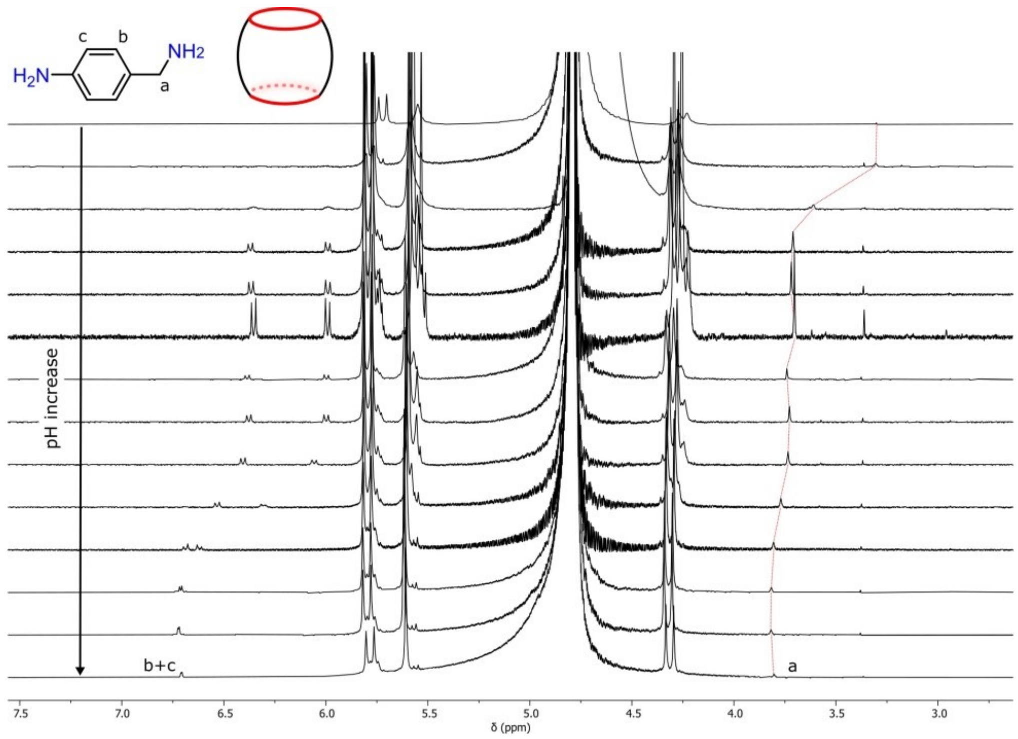

Fig. S11 ^{1}H NMR spectra of 4-aminobenzylamine in the presence of CB7 (4 equiv) at various pH_{obs} (D_2O, 298 K).

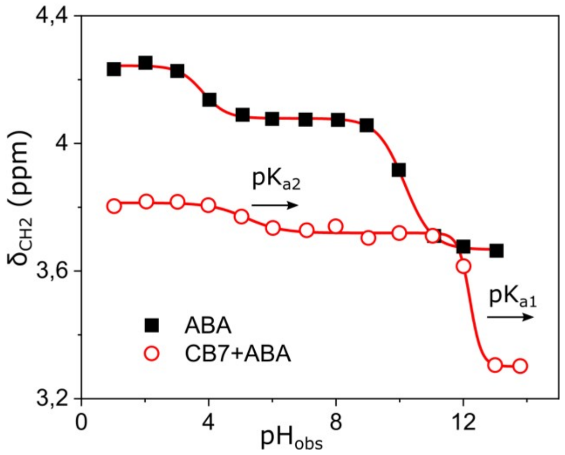

Fig. S12 Changes in chemical shifts of the methylene group in the presence and absence of CB7 depending on pH_{obs} (D_2O, 298 K).

6. Binding studies

Table S2. Binding thermodynamics of substrate with CB7.

N, (sites)	ΔH, kcal/mol	-TΔS, kcal/mol	ΔG, kcal/mol
0.95	-10.1±0.1	1.7	-8.38

Table S3. Binding thermodynamics of CB7 with AuMUS.

binding event	N, (sites)	ΔH, kcal/mol	-TΔS, kcal/mol	ΔG, kcal/mol
1	5.09	-11.6±0.2	2.95	-8.65
2	41.95	-3.07±0.04	-3.56	-6.63

Table S4. Binding thermodynamics of substrate with AuMUS.

N, (sites)	ΔH, kcal/mol	-TΔS, kcal/mol	ΔG, kcal/mol
28.90	-3.77±0.09	-3.48	-7.25

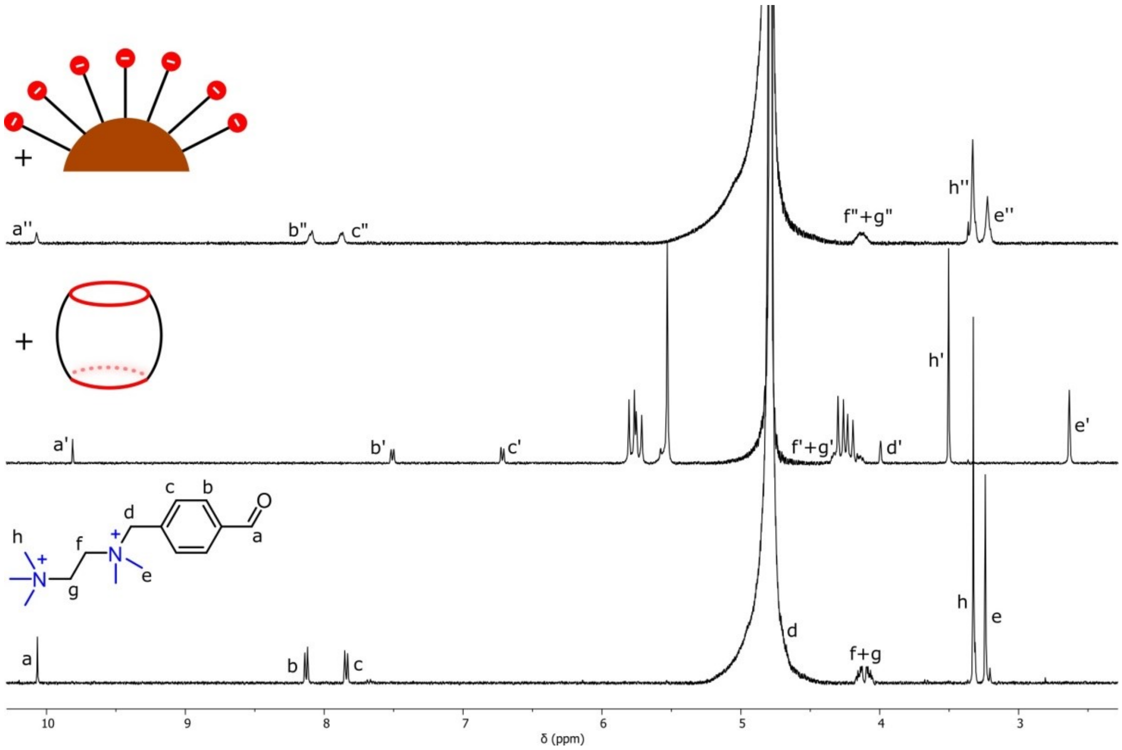

Fig. S13 ^{1}H NMR spectra obtained for the substrate alone and when combined with CB7 (1 equiv) and AuMUS (1 equiv) in D_2O at 298 K. The upfield shifts observed in the aromatic and aldehyde proton signals indicate the binding of the substrate within the CB7 cavity.

7. Kinetic studies

The advancement of the reaction was monitored by the emergence of a peak at a wavelength of 267 nm associated with the product. Measurements were conducted at 1 second intervals (or every 10 seconds for the uncatalyzed reaction).

The reaction rate was determined with respect to the product (oxime), on which the reaction shows a pseudo-first-order dependence due to a significant excess of hydroxylamine. The product yield, constrained to 40%, was calculated using the formula:

$$Y = \frac{A - A_0}{A_\infty - A_0}$$

where A is the absorbance at a time t, A_0 is the absorbance at the beginning of the reaction, and A_∞ is the absorbance at 100% conversion.

The rate constant (*k*) was calculated as the slope of the function:

$$ln\frac{1}{1 - Y} = kt$$

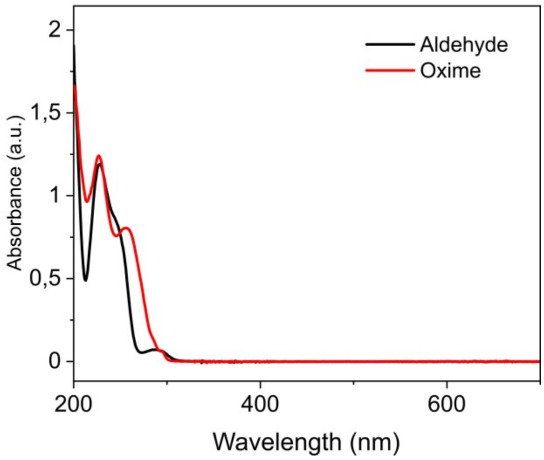

Fig. S14 Absorbance spectra for aldehyde (substrate) and oxime (product).

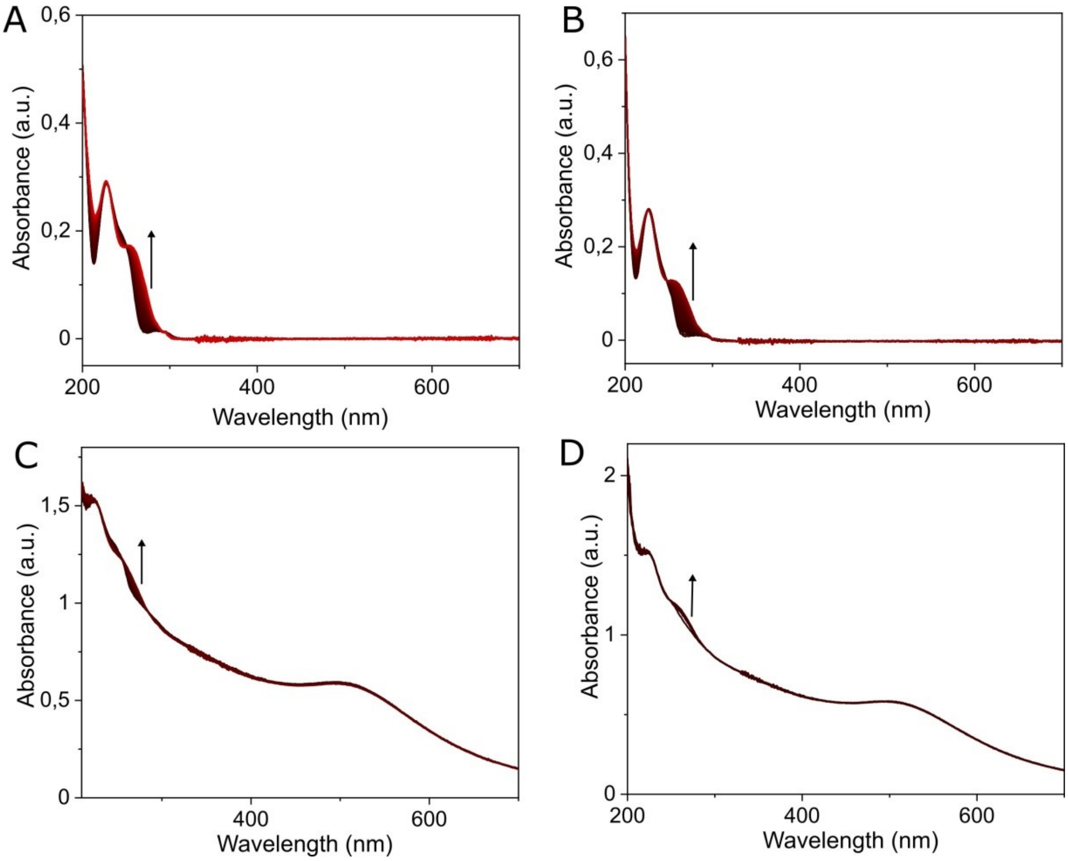

Fig. S15 Time-resolved absorbance spectra for the oxime formation reaction: (A) without catalyst; (B) CB7 (1 equiv); (C) AuMUS (4 equiv); (D) a mixture of CB7 (1 equiv) and AuMUS (4 equiv), D_2O, pH=6, 298 K.

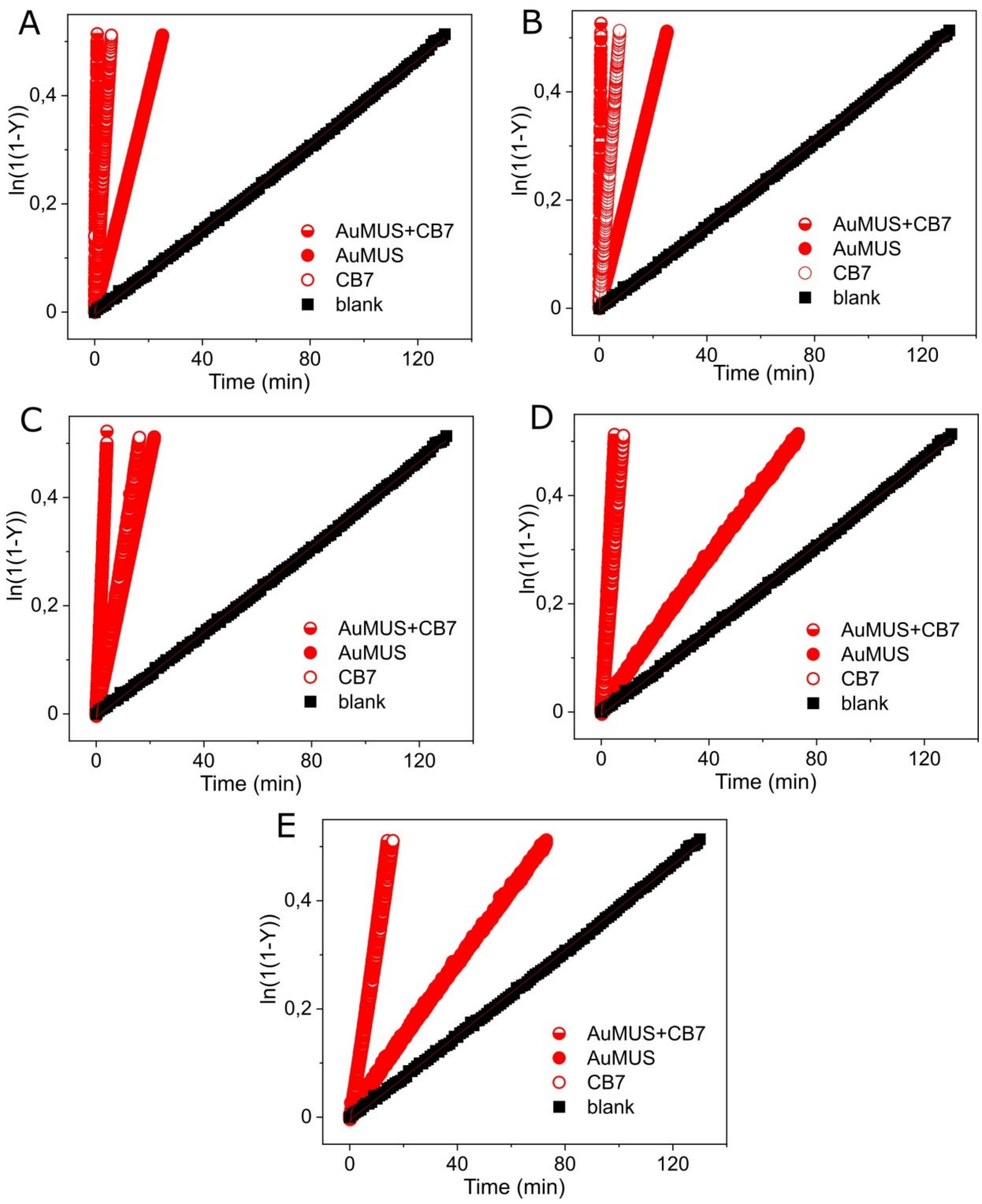

Fig. S16 Kinetic traces for the oxime formation reaction in the presence of catalysts used together and separately: (A) CB7 (4 equiv), AuMUS (1 equiv); (B) CB7 (1 equiv), AuMUS (1 equiv); (C) CB7 (0.25 equiv), AuMUS (1 equiv); (D) CB7 (1 equiv), AuMUS (0.25 equiv); (E) CB7 (0.25 equiv), AuMUS (0.25 equiv).

Table S5. Kinetic data for the oxime formation reaction in the presence of CB7 and AuMUS.

	no catalyst	CB7			AuMUS		
		0.25 equiv	1 equiv	4 equiv	0.25 equiv	1 equiv	4 equiv
k_{obs} (s^{-1})	0.0039	0.0322	0.0672	0.0820	0.0069	0.0203	0.0738
α	-	8.3	17.2	21.0	1.8	5.2	18.9

Table S6. Rate constants for the oxime formation reaction in the presence of the CB7-AuMUS mixture.

	CB7:AuMUS ratio					
	1:4	4:1	1:1	0.25:1	1:0.25	0.25:0.25
k_{obs} (s^{-1})	4.0701	0.5175	0.7842	0.1271	0.1027	0.0367

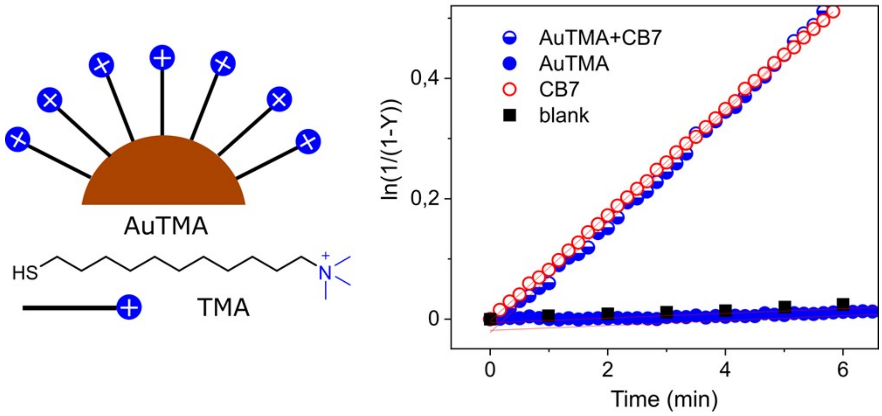

Fig. S17 Kinetic traces for the oxime formation reaction in the presence of CB7 (1 equiv) and AuTMA (1 equiv), used together and separately.

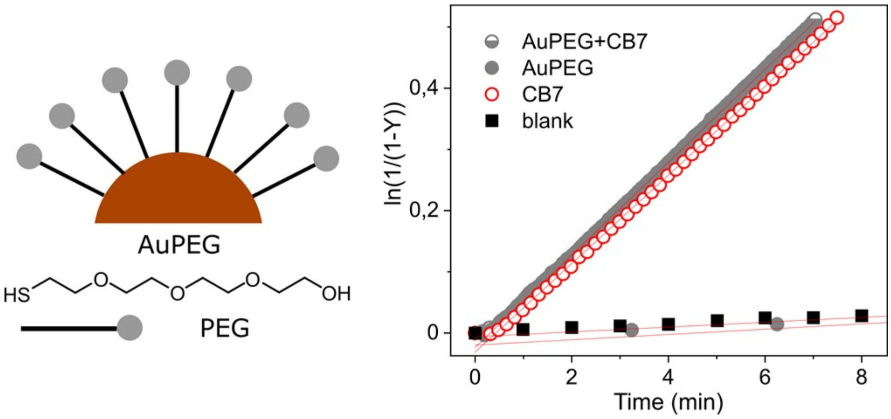

Fig. S18 Kinetic traces for the oxime formation reaction in the presence of CB7 (1 equiv) and AuPEG (1 equiv), used together and separately.

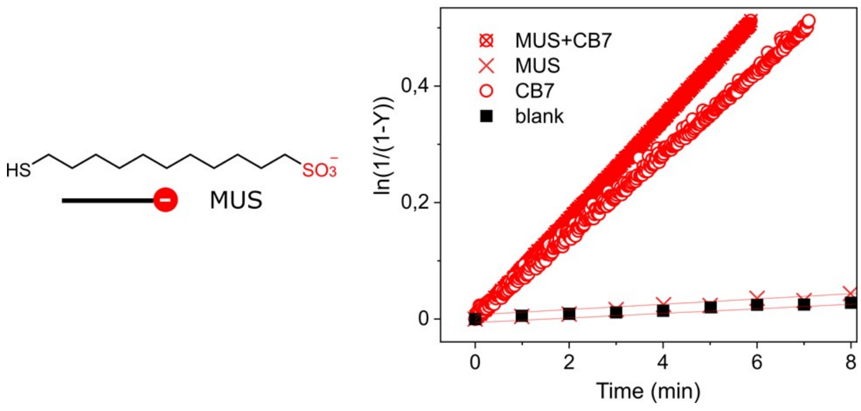

Fig. S19 Kinetic traces for the oxime formation reaction in the presence of CB7 (1 equiv) and MUS (1 equiv), used together and separately.

Table S7. Kinetic data for the oxime formation reaction in the presence of AuTMA, AuPEG and MUS.

	AuTMA	AuTMA+CB7	AuPEG	AuPEG+CB7	MUS	MUS+CB7
	1 equiv	1 equiv	1 equiv	1 equiv	1 equiv	1 equiv
k_{obs} (s^{-1})	0.0040	0.0917	0.0043	0.0757	0.0045	0.0869
α	1.03	23.5	1.1	19.4	1.15	22.3

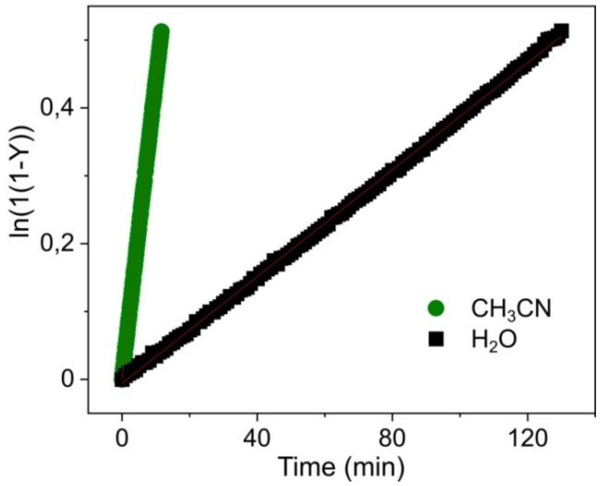

Fig. S20 Kinetic traces for the oxime formation reaction conducted separately in acetonitrile and water. The acetonitrile employed in the experiment contained 0.1% v/v hydroxylamine-containing water. The pH of the hydroxylamine-containing water was adjusted to 6 before its addition to the reaction system.

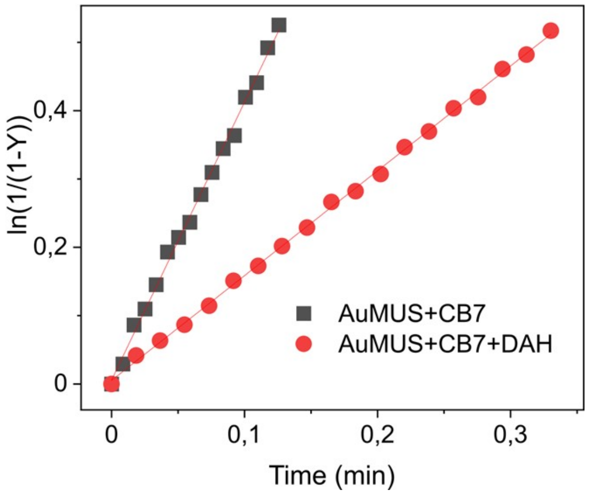

Fig. S21 Kinetic traces for the oxime formation reaction conducted in the absence and presence of 1 equivalent of 1,6-diaminohexane (DAH).

Table S8. Kinetic data for the oxime formation reaction conducted in acetonitrile.

	CH_3CN
k_{obs} (s^{-1})	0.0439
α	11.3

8. Computational results

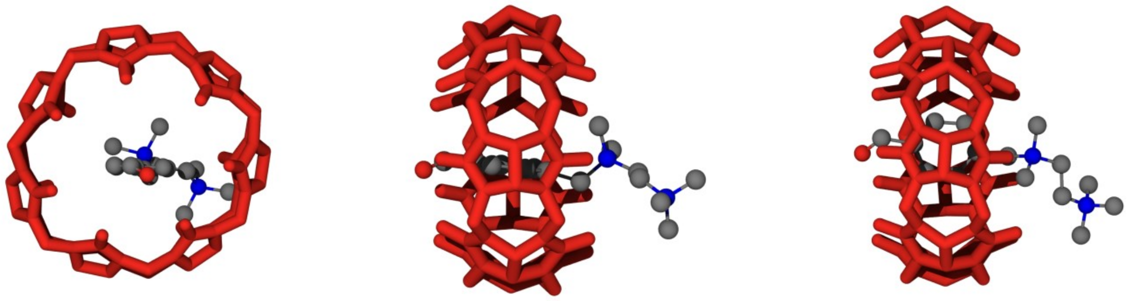

Fig. S22 Molecular structure (top and lateral views) of substrate ⊂ CB7 extracted from equilibrated atomistic simulations. CB7 molecule is colored in orange, while the atoms of the substrate are colored by element (C, grey; N, blue; O, red). Hydrogen atoms, water molecules, and counterions were removed for better clarity.

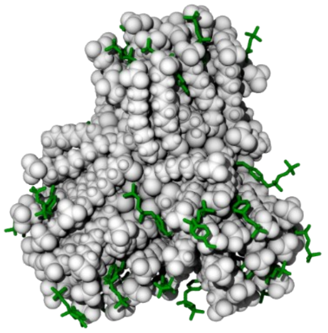

Fig. S23 Molecular structure of AuMUS nanoparticle with aldehydes interacting with the monolayer, extracted from equilibrated atomistic simulations. Atoms of ligands are colored in white, while aldehydes molecules are colored in green, with hydrogen atoms removed for better clarity. Water molecules and counterions are not shown.

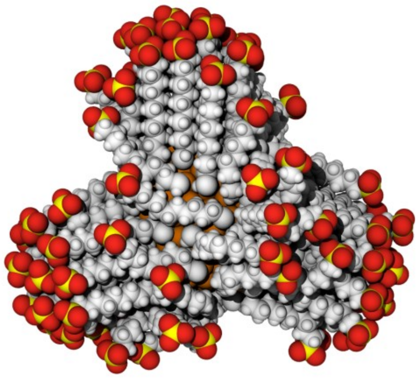

Fig. S24 Molecular structure of AuMUS nanoparticle extracted from equilibrated atomistic simulations, showing clustering of ligands on the gold surface. Water molecules and counterions were removed for better clarity. Atoms are colored by element (C, grey; O, red; S, yellow; H, white).

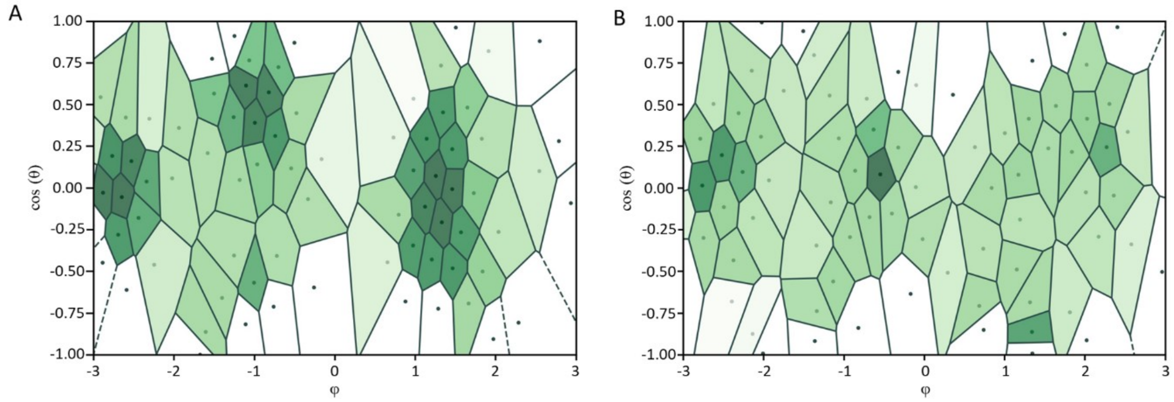

Fig. S25 Voronoi tessellation based on the center of mass (COM) of MUS ligands projected onto a bi-dimensional (ϕ, cos (θ)) plane for (A) AuMUS nanoparticle and (B) AuMUS nanoparticle after CB7 binding (after the monolayer's structural reconfiguration). Each polygon is colored according to its value, where darker (lighter) regions represent smaller (larger) area with higher (lower) ligand density with respect to the average.

Table S9. Binding free energy of CB7/substrate and AuMUS/CB7 (ΔG, kcal mol^{-1}) from restrained umbrella sampling calculations.

	CB7/substrate	AuMUS/CB7	
		CB7@B1	CB7@B2
ΔG	-8.5 ± 0.1	-10.6 ± 1.5	-6.4 ± 1.3

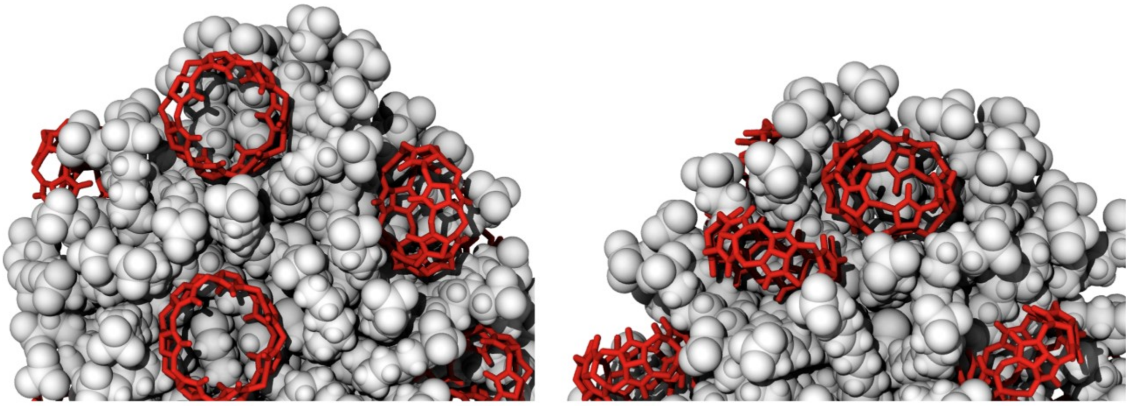

Fig. S26 Different zoom views of AuMUS (white)/CB7 (red) complex at high loading of CB7.

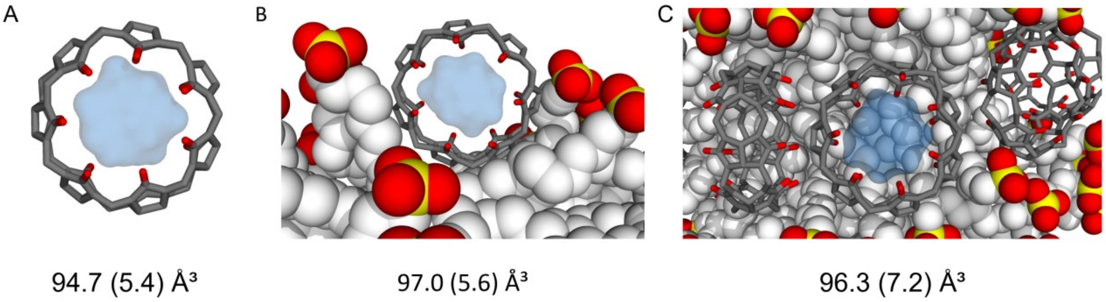

Fig. S27 Cavity volume of (A) CB7 in solution, (B) CB7@B1, and (C) CB7@B2. The cavity volume is shown as a light blue surface and the average value with standard deviation is reported. Calculation of the cavity volume was performed using CageCavityCalc (C3)[27] (probe radius 1Å).

9. Appendix

9.1 MD input files

Example of AMBER 22 minimization input file:

```
&cntrl
 ntpr = 1000,
 ioutfm = 1,
 ntxo = 1,
 iwrap = 0,
 cut = 10.0,
 imin = 1,
 maxcyc = 100000,
 ncyc = 1500,
 ntmin = 1,
 ntr = 1,
 restraint_wt = 200,
 restraintmask = '@SH,AU',
/
```

Example of AMBER 22 NVT simulation input file:

```
&cntrl
 ioutfm = 1,
 ntxo = 2,
 iwrap = 0,
 cut = 10.0,
 ntc = 2,
 tol = 0.000001,
 ntf = 2,
 ntpr = 500,
 ntwx = 1000,
 ntwr = 50000,
 nstlim = 100000,
 dt = 0.001,
 ig = -1,
 ntt = 3,
 gamma_ln = 1.0,
 temp0 = 300.0,
 ntr = 1,
 restraint_wt = 200,
 restraintmask = '@SH,AU',
/

&wt TYPE='TEMP0',
    value1=0.1, value2=300.0,
    istep1=0, istep2=100000,
    /
&wt TYPE='END'
/
```

Example of AMBER 22 NPT simulation input file:

```
&cntrl
 ioutfm = 1,
 ntxo = 2,
 iwrap = 0,
 cut = 10.0,
 irest = 1,
 ntc = 2,
 tol = 0.000001,
 ntf = 2,
 ntx = 5,
 ntb= 2,
 ntpr = 500000,
 ntwx = 50000,
 ntwr = 1000000,
 nstlim = 50000000,
 dt = 0.002,
 ig = -1,
 ntt = 3,
 gamma_ln = 2.0,
 temp0 = 300.0,
```

```
 ntp = 1,
 barostat = 2,
 taup = 1.2,
 ntr = 1,
 restraint_wt = 200
 restraintmask = '@SH,AU',
/
```

Example of AMBER 22 NPT simulation input file with umbrella sampling restraint:

```
&cntrl
 ioutfm = 1,
 ntxo = 2,
 iwrap = 0,
 cut = 10.0,
 irest = 1,
 ntc = 2,
 tol = 0.000001,
 ntf = 2,
 ntx = 5,
 ntb= 2,
 ntpr = 10000,
 ntwx = 10000,
 ntwr = 50000,
 nstlim = 750000,
 dt = 0.002,
 ig = -1,
 ntt = 3,
 gamma_ln = 2.0,
 temp0 = 300.0,
 ntp = 1,
 barostat = 2,
 taup = 1.2,
 ntr = 1,
 nmropt = 0,
 infe=1,
/
&pmd
 output_file='collective_variable.dat'
 output_freq=1000
 cv_file='US_restraint.in'
/
&end
&wt
 type='END',
&end
```

Example of AMBER 22 umbrella sampling restraint file

```
&colvar
  cv_type='DISTANCE'
  cv_ni=2  cv_i=171,16
  anchor_position=0,2.85,2.85,102.85
  anchor_strength=10.0,10.0
/
```

9.2 MD molecular starting structures

Starting structure of MUS ligand reported in mol2 format:

```
@<TRIPOS>MOLECULE
MUS
   39    38     1     0     0
SMALL
```

```
USER_CHARGES

@<TRIPOS>ATOM
      1 SH         -12.5490     6.7780     1.4960 sh         1 MUS       -0.388900
      2 HS         -13.4080     7.5610     0.8110 hs         1 MUS        0.209700
      3 C1         -11.1460     6.8230     0.2890 c3         1 MUS       -0.159300
      4 H11        -11.5190     6.4370    -0.6680 h1         1 MUS        0.096900
      5 H12        -10.8640     7.8750     0.1490 h1         1 MUS        0.096900
      6 C2          -9.9180     5.9990     0.7560 c3         1 MUS        0.016700
      7 H21         -9.5890     6.3890     1.7280 hc         1 MUS        0.062000
      8 H22        -10.2400     4.9630     0.9230 hc         1 MUS        0.062000
      9 C3          -8.7210     6.0140    -0.2390 c3         1 MUS       -0.035900
     10 H31         -9.0550     5.6180    -1.2070 hc         1 MUS        0.012800
     11 H32         -8.4100     7.0530    -0.4100 hc         1 MUS        0.012800
     12 C4          -7.5010     5.1890     0.2620 c3         1 MUS       -0.001100
     13 H41         -7.1620     5.5920     1.2240 hc         1 MUS       -0.009900
     14 H42         -7.8140     4.1540     0.4450 hc         1 MUS       -0.009900
     15 C5          -6.3030     5.1790    -0.7250 c3         1 MUS        0.034700
     16 H51         -5.9720     6.2060    -0.9200 hc         1 MUS       -0.011200
     17 H52         -6.6170     4.7560    -1.6860 hc         1 MUS       -0.011200
     18 C6          -5.0980     4.3580    -0.1840 c3         1 MUS        0.023000
     19 H61         -5.4110     3.3270     0.0190 hc         1 MUS       -0.005600
     20 H62         -4.7510     4.7870     0.7650 hc         1 MUS       -0.005600
     21 C7          -3.9150     4.3300    -1.1810 c3         1 MUS       -0.001400
     22 H71         -3.5960     5.3590    -1.4000 hc         1 MUS       -0.011600
     23 H72         -4.2500     3.8880    -2.1300 hc         1 MUS       -0.011600
     24 C8          -2.6930     3.5260    -0.6590 c3         1 MUS        0.011400
     25 H81         -3.0080     2.5000    -0.4210 hc         1 MUS       -0.009200
     26 H82         -2.3370     3.9760     0.2790 hc         1 MUS       -0.009200
     27 C9          -1.5670     3.5190    -1.7240 c3         1 MUS        0.034700
     28 H91         -1.3140     4.5620    -1.9460 hc         1 MUS       -0.015800
     29 H92         -1.9810     3.0860    -2.6420 hc         1 MUS       -0.015800
     30 C10         -0.3010     2.7400    -1.2790 c3         1 MUS        0.043600
     31 H01          0.0780     3.1920    -0.3610 hc         1 MUS        0.009500
     32 H02         -0.5890     1.7140    -1.0420 hc         1 MUS        0.009500
     33 C11          0.8160     2.7310    -2.3530 c3         1 MUS       -0.190300
     34 H13          1.1720     3.7380    -2.5790 h1         1 MUS        0.051400
     35 H14          0.4900     2.2490    -3.2750 h1         1 MUS        0.051400
     36 SO           2.2920     1.8330    -1.8820 s6         1 MUS        1.163200
     37 O1           2.7210     2.5220    -0.6800 o          1 MUS       -0.696200
     38 O2           1.7880     0.4910    -1.6770 o          1 MUS       -0.696200
     39 O3           3.1470     2.0010    -3.0400 o          1 MUS       -0.706300
@<TRIPOS>BOND
     1      1      2 1
     2      1      3 1
     3      3      4 1
     4      3      5 1
     5      3      6 1
     6      6      7 1
     7      6      8 1
     8      6      9 1
     9      9     10 1
    10      9     11 1
    11      9     12 1
    12     12     13 1
    13     12     14 1
    14     12     15 1
    15     15     16 1
    16     15     17 1
    17     15     18 1
    18     18     19 1
    19     18     20 1
    20     18     21 1
    21     21     22 1
    22     21     23 1
    23     21     24 1
    24     24     25 1
    25     24     26 1
    26     24     27 1
    27     27     28 1
    28     27     29 1
```

```
     29    27    30 1
     30    30    31 1
     31    30    32 1
     32    30    33 1
     33    33    34 1
     34    33    35 1
     35    33    36 1
     36    36    37 1
     37    36    38 1
     38    36    39 1
@<TRIPOS>SUBSTRUCTURE
     1 MUS         1 TEMP              0 ****  ****    0 ROOT
```

Starting structure of CB7 molecule reported in mol2 format:

```
@<TRIPOS>MOLECULE
CB7
  126   147     1     0     0
SMALL
USER_CHARGES


@<TRIPOS>ATOM
      1 N1          -23.7320    15.7530     6.8890 n          1 CB7       -0.0999
      2 C1          -22.7640    16.4010     6.0600 c3         1 CB7       -0.0839
      3 C2          -22.5200    15.4000     4.8950 c3         1 CB7       -0.0839
      4 N2          -21.4630    16.6180     6.6160 n          1 CB7       -0.0999
      5 N3          -23.3810    14.2980     5.1950 n          1 CB7       -0.0999
      6 N4          -21.1140    15.1610     4.9240 n          1 CB7       -0.0999
      7 C3          -23.6740    13.2290     4.2830 c3         1 CB7        0.0395
      8 C4          -24.4360    16.3910     7.9620 c3         1 CB7        0.0395
      9 C5          -24.1480    14.5500     6.3280 c          1 CB7        0.4174
     10 C6          -20.3860    14.4770     3.8940 c3         1 CB7        0.0395
     11 C7          -21.1520    17.6440     7.5690 c3         1 CB7        0.0395
     12 C8          -20.4880    15.9420     5.8920 c          1 CB7        0.4174
     13 O1          -25.1000    13.8880     6.6970 o          1 CB7       -0.4185
     14 O2          -19.2860    16.1020     6.0050 o          1 CB7       -0.4185
     15 N5          -22.6920    12.1840     4.2500 n          1 CB7       -0.0999
     16 N6          -20.4220    13.0460     3.9800 n          1 CB7       -0.0999
     17 C9          -21.4860    12.2330     3.4810 c3         1 CB7       -0.0839
     18 C10         -22.9540    10.8920     4.6920 c          1 CB7        0.4174
     19 C11         -19.2940    12.2790     4.2590 c          1 CB7        0.4174
     20 O3          -18.1820    12.7200     4.4980 o          1 CB7       -0.4185
     21 N7          -19.6100    10.9370     4.0810 n          1 CB7       -0.0999
     22 O4          -24.0010    10.5120     5.1880 o          1 CB7       -0.4185
     23 N8          -21.8810    10.0770     4.3500 n          1 CB7       -0.0999
     24 C12         -20.9280    10.7830     3.5490 c3         1 CB7       -0.0839
     25 C13         -18.6220     9.8990     4.1100 c3         1 CB7        0.0395
     26 C14         -21.9080     8.6520     4.5010 c3         1 CB7        0.0395
     27 N9          -21.0070     8.1340     5.4880 n          1 CB7       -0.0999
     28 N10         -18.7370     8.9930     5.2170 n          1 CB7       -0.0999
     29 N11         -23.7180    16.4460     9.2040 n          1 CB7       -0.0999
     30 N12         -21.4490    17.3100     8.9300 n          1 CB7       -0.0999
     31 C15         -19.6200     7.8710     5.2540 c3         1 CB7       -0.0839
     32 C16         -17.7810     8.9140     6.2230 c          1 CB7        0.4174
     33 C17         -21.4420     7.5290     6.6610 c          1 CB7        0.4174
     34 O5          -16.7850     9.6120     6.3120 o          1 CB7       -0.4185
     35 N13         -18.0740     7.8230     7.0340 n          1 CB7       -0.0999
     36 O6          -22.6040     7.4080     7.0060 o          1 CB7       -0.4185
     37 N14         -20.3440     6.9630     7.3040 n          1 CB7       -0.0999
     38 C18         -19.1670     7.0650     6.5030 c3         1 CB7       -0.0839
     39 C19         -22.7430    17.4380     9.5280 c3         1 CB7       -0.0839
     40 C20         -24.1210    15.7470    10.3370 c          1 CB7        0.4174
     41 C21         -20.4650    17.1410     9.8960 c          1 CB7        0.4174
     42 O7          -19.2630    17.2100     9.7030 o          1 CB7       -0.4185
     43 N15         -21.0740    17.0190    11.1400 n          1 CB7       -0.0999
     44 O8          -25.0770    14.9910    10.4000 o          1 CB7       -0.4185
     45 N16         -23.3420    16.1540    11.4120 n          1 CB7       -0.0999
```

46	C22	-22.4840	17.2400	11.0480	c3	1	CB7	-0.0839
47	N17	-20.3640	15.7690	13.0830	n	1	CB7	-0.0999
48	N18	-22.6330	14.9040	13.3560	n	1	CB7	-0.0999
49	C23	-19.2370	14.9740	13.2570	c	1	CB7	0.4174
50	C24	-21.4210	15.3650	13.9580	c3	1	CB7	-0.0839
51	C25	-22.8950	13.5850	13.6990	c	1	CB7	0.4174
52	O9	-23.9460	12.9960	13.5070	o	1	CB7	-0.4185
53	N19	-21.8150	13.0890	14.4190	n	1	CB7	-0.0999
54	O10	-18.1300	15.2070	12.8000	o	1	CB7	-0.4185
55	N20	-19.5450	13.9490	14.1440	n	1	CB7	-0.0999
56	C26	-21.8400	11.8120	15.0770	c3	1	CB7	0.0395
57	C27	-20.8560	14.1160	14.6900	c3	1	CB7	-0.0839
58	C28	-18.5530	13.0610	14.6780	c3	1	CB7	0.0395
59	N21	-20.9480	10.8360	14.5260	n	1	CB7	-0.0999
60	N22	-18.6770	11.6970	14.2520	n	1	CB7	-0.0999
61	C29	-21.3940	9.6870	13.8830	c	1	CB7	0.4174
62	C30	-19.6080	10.7910	14.8120	c3	1	CB7	-0.0839
63	C31	-17.7340	11.0750	13.4390	c	1	CB7	0.4174
64	O11	-16.7430	11.6070	12.9710	o	1	CB7	-0.4185
65	N23	-18.0330	9.7180	13.3680	n	1	CB7	-0.0999
66	O12	-22.5590	9.3990	13.6780	o	1	CB7	-0.4185
67	N24	-20.3020	8.8570	13.6440	n	1	CB7	-0.0999
68	C32	-17.1540	8.7630	12.7610	c3	1	CB7	0.0395
69	C33	-20.4400	7.5160	13.1570	c3	1	CB7	0.0395
70	C34	-19.1140	9.3820	14.2410	c3	1	CB7	-0.0839
71	N25	-19.9300	7.3000	11.8340	n	1	CB7	-0.0999
72	N26	-17.6570	8.1600	11.5600	n	1	CB7	-0.0999
73	C35	-20.7470	6.9930	10.7520	c	1	CB7	0.4174
74	C36	-18.5560	7.0470	11.5320	c3	1	CB7	-0.0839
75	C37	-17.0880	8.3770	10.3120	c	1	CB7	0.4174
76	O13	-16.1450	9.1150	10.0840	o	1	CB7	-0.4185
77	N27	-17.6730	7.5130	9.3940	n	1	CB7	-0.0999
78	O14	-21.9630	6.9140	10.7840	o	1	CB7	-0.4185
79	N28	-19.9440	6.6550	9.6680	n	1	CB7	-0.0999
80	C38	-18.5640	6.5990	10.0420	c3	1	CB7	-0.0839
81	C39	-20.3340	17.0140	12.3690	c3	1	CB7	0.0395
82	C40	-23.6200	15.7630	12.7640	c3	1	CB7	0.0395
83	C41	-20.4740	6.1100	8.4500	c3	1	CB7	0.0395
84	C42	-17.1870	7.3530	8.0560	c3	1	CB7	0.0395
85	H1	-23.1730	17.3960	5.8290	h2	1	CB7	0.1165
86	H2	-22.7580	15.7090	3.8670	h2	1	CB7	0.1165
87	H3	-23.7410	13.6590	3.2730	h2	1	CB7	0.0644
88	H4	-24.6310	12.7810	4.5880	h2	1	CB7	0.0644
89	H5	-24.6520	17.4250	7.6540	h2	1	CB7	0.0644
90	H6	-25.3640	15.8270	8.1360	h2	1	CB7	0.0644
91	H7	-19.3340	14.7900	3.9630	h2	1	CB7	0.0644
92	H8	-20.8230	14.7650	2.9270	h2	1	CB7	0.0644
93	H9	-20.0730	17.8480	7.5020	h2	1	CB7	0.0644
94	H10	-21.7410	18.5350	7.3060	h2	1	CB7	0.0644
95	H11	-21.7600	12.6520	2.5020	h2	1	CB7	0.1165
96	H12	-20.8200	10.2090	2.6170	h2	1	CB7	0.1165
97	H13	-18.7230	9.3130	3.1850	h2	1	CB7	0.0644
98	H14	-17.6330	10.3780	4.1690	h2	1	CB7	0.0644
99	H15	-22.9280	8.3660	4.7960	h2	1	CB7	0.0644
100	H16	-21.6350	8.2080	3.5330	h2	1	CB7	0.0644
101	H17	-19.5570	7.3970	4.2640	h2	1	CB7	0.1165
102	H18	-18.7960	6.0380	6.3680	h2	1	CB7	0.1165
103	H19	-23.1510	18.3970	9.1770	h2	1	CB7	0.1165
104	H20	-22.7140	18.0640	11.7390	h2	1	CB7	0.1165
105	H21	-21.6890	16.2530	14.5490	h2	1	CB7	0.1165
106	H22	-21.5560	11.9710	16.1280	h2	1	CB7	0.0644
107	H23	-22.8620	11.4140	14.9980	h2	1	CB7	0.0644
108	H24	-20.7330	14.1480	15.7830	h2	1	CB7	0.1165
109	H25	-17.5670	13.4250	14.3540	h2	1	CB7	0.0644
110	H26	-18.6420	13.0790	15.7740	h2	1	CB7	0.0644
111	H27	-19.5970	11.0240	15.8870	h2	1	CB7	0.1165
112	H28	-16.9770	7.9590	13.4910	h2	1	CB7	0.0644
113	H29	-16.2180	9.2830	12.5080	h2	1	CB7	0.0644
114	H30	-21.5130	7.2720	13.1460	h2	1	CB7	0.0644
115	H31	-19.8890	6.8530	13.8400	h2	1	CB7	0.0644
116	H32	-18.7220	8.6160	14.9270	h2	1	CB7	0.1165
117	H33	-18.1990	6.3440	12.2990	h2	1	CB7	0.1165

```
    118 H34         -18.2120     5.5890     9.7870 h2         1 CB7         0.1165
    119 H35         -19.2840    17.2380    12.1290 h2         1 CB7         0.0644
    120 H36         -20.7650    17.7860    13.0230 h2         1 CB7         0.0644
    121 H37         -23.6770    16.6770    13.3730 h2         1 CB7         0.0644
    122 H38         -24.5790    15.2240    12.7670 h2         1 CB7         0.0644
    123 H39         -21.5460     5.9190     8.6060 h2         1 CB7         0.0644
    124 H40         -19.9320     5.1770     8.2370 h2         1 CB7         0.0644
    125 H41         -17.0200     6.2790     7.8860 h2         1 CB7         0.0644
    126 H42         -16.2480     7.9190     7.9710 h2         1 CB7         0.0644
@<TRIPOS>BOND
     1     1     2 1
     2     1     8 1
     3     1     9 1
     4     2     3 1
     5     2     4 1
     6     2    85 1
     7     3     5 1
     8     3     6 1
     9     3    86 1
    10     4    11 1
    11     4    12 1
    12     5     7 1
    13     5     9 1
    14     6    10 1
    15     6    12 1
    16     7    15 1
    17     7    87 1
    18     7    88 1
    19     8    29 1
    20     8    89 1
    21     8    90 1
    22     9    13 2
    23    10    16 1
    24    10    91 1
    25    10    92 1
    26    11    30 1
    27    11    93 1
    28    11    94 1
    29    12    14 2
    30    15    17 1
    31    15    18 1
    32    16    17 1
    33    16    19 1
    34    17    24 1
    35    17    95 1
    36    18    22 2
    37    18    23 1
    38    19    20 2
    39    19    21 1
    40    21    24 1
    41    21    25 1
    42    23    24 1
    43    23    26 1
    44    24    96 1
    45    25    28 1
    46    25    97 1
    47    25    98 1
    48    26    27 1
    49    26    99 1
    50    26   100 1
    51    27    31 1
    52    27    33 1
    53    28    31 1
    54    28    32 1
    55    29    39 1
    56    29    40 1
    57    30    39 1
    58    30    41 1
    59    31    38 1
    60    31   101 1
    61    32    34 2
    62    32    35 1
```

63	33	36	2
64	33	37	1
65	35	38	1
66	35	84	1
67	37	38	1
68	37	83	1
69	38	102	1
70	39	46	1
71	39	103	1
72	40	44	2
73	40	45	1
74	41	42	2
75	41	43	1
76	43	46	1
77	43	81	1
78	45	46	1
79	45	82	1
80	46	104	1
81	47	49	1
82	47	50	1
83	47	81	1
84	48	50	1
85	48	51	1
86	48	82	1
87	49	54	2
88	49	55	1
89	50	57	1
90	50	105	1
91	51	52	2
92	51	53	1
93	53	56	1
94	53	57	1
95	55	57	1
96	55	58	1
97	56	59	1
98	56	106	1
99	56	107	1
100	57	108	1
101	58	60	1
102	58	109	1
103	58	110	1
104	59	61	1
105	59	62	1
106	60	62	1
107	60	63	1
108	61	66	2
109	61	67	1
110	62	70	1
111	62	111	1
112	63	64	2
113	63	65	1
114	65	68	1
115	65	70	1
116	67	69	1
117	67	70	1
118	68	72	1
119	68	112	1
120	68	113	1
121	69	71	1
122	69	114	1
123	69	115	1
124	70	116	1
125	71	73	1
126	71	74	1
127	72	74	1
128	72	75	1
129	73	78	2
130	73	79	1
131	74	80	1
132	74	117	1
133	75	76	2
134	75	77	1

```
   135    77    80 1
   136    77    84 1
   137    79    80 1
   138    79    83 1
   139    80   118 1
   140    81   119 1
   141    81   120 1
   142    82   121 1
   143    82   122 1
   144    83   123 1
   145    83   124 1
   146    84   125 1
   147    84   126 1
@<TRIPOS>SUBSTRUCTURE
     1 CB7         1 TEMP              0 ****  ****    0 ROOT
```

Starting structure of aldehyde molecule reported in mol2 format:

```
@<TRIPOS>MOLECULE
ALD
   44    44     1     0     0
SMALL
USER_CHARGES


@<TRIPOS>ATOM
      1 C4         -1.4980    -0.1510     0.0360 ca         1 ALD      -0.0956
      2 H3         -2.5320    -0.0580    -0.1640 ha         1 ALD       0.1184
      3 C5         -0.9300    -1.4200     0.1530 ca         1 ALD       0.0254
      4 C6          0.4440    -1.5050     0.4190 ca         1 ALD      -0.0956
      5 H4          0.9130    -2.4490     0.5150 ha         1 ALD       0.1184
      6 C7          1.2130    -0.3470     0.5600 ca         1 ALD      -0.1130
      7 H5          2.2690    -0.4080     0.7630 ha         1 ALD       0.1493
      8 C2          0.6240     0.9050     0.4380 ca         1 ALD       0.0412
      9 C3         -0.7340     1.0010     0.1770 ca         1 ALD      -0.1130
     10 H2         -1.1890     1.9560     0.0830 ha         1 ALD       0.1493
     11 C1          1.4370     2.1120     0.5860 c          1 ALD       0.3520
     12 H1          0.9940     3.0770     0.4940 h4         1 ALD       0.0276
     13 O1          2.6430     2.0020     0.8160 o          1 ALD      -0.3282
     14 C8         -1.9390    -2.6450    -0.0380 c3         1 ALD      -0.1270
     15 H7         -2.3470    -2.5390    -1.0580 hx         1 ALD       0.1294
     16 H6         -2.7890    -2.4380     0.6340 hx         1 ALD       0.1294
     17 N1         -1.5220    -4.1110     0.1530 n4         1 ALD       0.0442
     18 C11        -1.0720    -4.3730     1.5440 c3         1 ALD      -0.0543
     19 H11        -0.8280    -5.4420     1.6570 hx         1 ALD       0.0964
     20 H12        -0.1800    -3.7720     1.7680 hx         1 ALD       0.0964
     21 H10        -1.8770    -4.1080     2.2480 hx         1 ALD       0.0964
     22 C10        -2.7160    -4.9400    -0.1040 c3         1 ALD      -0.0450
     23 H9         -3.5150    -4.6660     0.6040 hx         1 ALD       0.0917
     24 H8         -3.0680    -4.7700    -1.1340 hx         1 ALD       0.0917
     25 C9         -0.4710    -4.5240    -0.8150 c3         1 ALD      -0.0543
     26 H15         0.4400    -3.9330    -0.6550 hx         1 ALD       0.0964
     27 H14        -0.2400    -5.5920    -0.6710 hx         1 ALD       0.0964
     28 H13        -0.8340    -4.3660    -1.8430 hx         1 ALD       0.0964
     29 C12        -2.3640    -6.4280     0.0760 c3         1 ALD      -0.1119
     30 H16        -1.8970    -6.7950    -0.8140 hx         1 ALD       0.1262
     31 H17        -1.6920    -6.5380     0.9020 hx         1 ALD       0.1262
     32 N2         -3.6490    -7.2310     0.3500 n4         1 ALD       0.0186
     33 C13        -4.8190    -6.2740     0.5120 c3         1 ALD      -0.1594
     34 H18        -5.5040    -6.4080    -0.2990 hx         1 ALD       0.1443
     35 H19        -5.3180    -6.4740     1.4370 hx         1 ALD       0.1443
     36 H20        -4.4580    -5.2670     0.5120 hx         1 ALD       0.1443
     37 C14        -3.9190    -8.1680    -0.8160 c3         1 ALD      -0.1594
     38 H21        -3.0010    -8.3780    -1.3240 hx         1 ALD       0.1443
     39 H22        -4.3420    -9.0800    -0.4500 hx         1 ALD       0.1443
     40 H23        -4.6040    -7.7050    -1.4950 hx         1 ALD       0.1443
     41 C15        -3.4780    -8.0430     1.6230 c3         1 ALD      -0.1594
     42 H24        -3.9010    -9.0160     1.4850 hx         1 ALD       0.1443
     43 H25        -2.4370    -8.1350     1.8510 hx         1 ALD       0.1443
     44 H26        -3.9770    -7.5490     2.4310 hx         1 ALD       0.1443
```

```
@<TRIPOS>BOND
     1     1     2 1
     2     1     3 ar
     3     1     9 ar
     4     3     4 ar
     5     3    14 1
     6     4     5 1
     7     4     6 ar
     8     6     7 1
     9     6     8 ar
    10     8     9 ar
    11     8    11 1
    12     9    10 1
    13    11    12 1
    14    11    13 2
    15    14    15 1
    16    14    16 1
    17    14    17 1
    18    17    18 1
    19    17    22 1
    20    17    25 1
    21    18    19 1
    22    18    20 1
    23    18    21 1
    24    22    23 1
    25    22    24 1
    26    22    29 1
    27    25    26 1
    28    25    27 1
    29    25    28 1
    30    29    30 1
    31    29    31 1
    32    29    32 1
    33    32    33 1
    34    32    37 1
    35    32    41 1
    36    33    34 1
    37    33    35 1
    38    33    36 1
    39    37    38 1
    40    37    39 1
    41    37    40 1
    42    41    42 1
    43    41    43 1
    44    41    44 1
@<TRIPOS>SUBSTRUCTURE
     1  ALD         1 TEMP              0 ****  ****    0 ROOT
```

10. References

(1) Larsen, A. H.; Mortensen, J. J.; Blomqvist, J.; Castelli, I. E.; Christensen, R.; Dułak, M.; Friis, J.; Groves, M. N.; Hammer, B.; Hargus, C.; Hermes, E. D.; Jennings, P. C.; Jensen, P. B.; Kermode, J.; Kitchin, J. R.; Kolsbjerg, E. L.; Kubal, J.; Kaasbjerg, K.; Lysgaard, S.; Maronsson, J. B.; Maxson, T.; Olsen, T.; Pastewka, L.; Peterson, A.; Rostgaard, C.; Schiøtz, J.; Schütt, O.; Strange, M.; Thygesen, K. S.; Vegge, T.; Vilhelmsen, L.; Walter, M.; Zeng, Z.; Jacobsen, K. W., The Atomic Simulation Environment—A Python library for working with atoms. *J. Phys.: Condens. Matter* **2017**, *29*, 273002.

(2) Klein, C.; Sallai, J.; Jones, T. J.; Iacovella, C. R.; McCabe, C.; Cummings, P. T. A Hierarchical, Component Based Approach to Screening Properties of Soft Matter. In Foundations of Molecular Modeling and Simulation. Molecular Modeling and Simulation (Applications and Perspectives); Snurr, R. Q., Adjiman, C. S., Kofke, D. A., Eds.; *Springer*, Singapore, **2016**; pp 79-92.

(3) Heinz, H.; Lin, T.-J.; Kishore Mishra, R.; Emami, F. S. Thermodynamically consistent force fields for the assembly of inorganic, organic, and biological nanostructures: The INTERFACE force field. *Langmuir* **2013**, *29*, 1754-1765.

(4) He, X.; Man, V. H.; Yang, W.; Lee, T.-S.; Wang, J. A fast and high-quality charge model for the next generation general AMBER force field. *J. Chem. Phys.* **2020**, *153*, 114502.

(5) Vanquelef, E.; Simon, S.; Marquant, G.; Garcia, E.; Klimerak, G.; Delepine, J. C.; Cieplak, P.; Dupradeau, F.-Y., R.E.D. Server: a web service for deriving RESP and ESP charges and building force field libraries for new molecules and molecular fragments, *Nucl. Acids Res.* (Web server issue) **2011**, 39, W511-W517.

(6) Wang, F.; Becker, J.-P.; Cieplak, P.; Dupradeau, F.-Y., R.E.D. Python: Object oriented programming for Amber force fields, Université de Picardie - Jules Verne, Sanford Burnham Prebys Medical Discovery Institute, Nov. **2013.**

(7) Dupradeau, F.-Y.; Pigache, A.; Zaffran, T.; Savineau, C.; Lelong, R; Grivel, N.; Lelong, D.; Rosanski, W.; Cieplak, P., The R.E.D. tools: Advances in RESP and ESP charge derivation and force field library building, *Phys. Chem. Chem. Phys.* **2010**, 12, 7821-7839.

(8) Bayly, C. I.; Cieplak, P.; Cornell, W.; Kollman, P. A., A well-behaved electrostatic potential based method using charge restraints for deriving atomic charges: the RESP model. *J. Phys. Chem.* **1993**, 97, 10269-10280.

(9) Gaussian 16, Revision C.01, Frisch, M. J.; Trucks, G. W.; Schlegel, H. B.; Scuseria, G. E.; Robb, M. A.; Cheeseman, J. R.; Scalmani, G.; Barone, V.; Petersson, G. A.; Nakatsuji, H.; Li, X.; Caricato, M.; Marenich, A. V.; Bloino, J.; Janesko, B. G.; Gomperts, R.; Mennucci, B.; Hratchian, H. P.; Ortiz, J. V.; Izmaylov, A. F.; Sonnenberg, J. L.; Williams-Young, D.; Ding, F.; Lipparini, F.; Egidi, F.; Goings, J.; Peng, B.; Petrone, A.; Henderson, T.; Ranasinghe, D.; Zakrzewski, V. G.; Gao, J.; Rega, N.; Zheng, G.; Liang, W.; Hada, M.; Ehara, M.; Toyota, K.; Fukuda, R.; Hasegawa, J.; Ishida, M.; Nakajima, T.; Honda, Y.; Kitao, O.; Nakai, H.; Vreven, T.; Throssell, K.; Montgomery, J. A., Jr.; Peralta, J. E.; Ogliaro, F.; Bearpark, M. J.; Heyd, J. J.; Brothers, E. N.; Kudin, K. N.; Staroverov, V. N.; Keith, T. A.; Kobayashi, R.; Normand, J.; Raghavachari, K.; Rendell, A. P.; Burant, J. C.; Iyengar, S. S.; Tomasi, J.; Cossi, M.; Millam, J. M.; Klene, M.; Adamo, C.; Cammi, R.; Ochterski, J. W.; Martin, R. L.; Morokuma, K.; Farkas, O.; Foresman, J. B.; Fox, D. J. Gaussian, Inc., Wallingford CT, 2016.

(10) Case, D. A.; Ben-Shalom, I. Y.; Brozell, S. R.; Cerutti, D. S.; Cheatham, I. T. E.; Cruzeiro, L V. W. D.; Darden, T. A.; Duke, R. E.; Ghoreishi, D.; Gilson, M. K.; H. Gohlke, H.; Goetz, A. W.; Greene, D.; Harris, R.; Homeyer, N.; Izadi, S.; Kovalenko, A.; Kurtzman, T.; Lee, T. S.; LeGrand, S.; Li, P.; Lin, C.; Liu, J.; Luchko, T.; Luo, R.; Mermelstein, D. J.; Merz, K. M.; Miao, Y.; Monard, G.; Nguyen, C.; Nguyen, H.; Omelyan, I.; Onufriev, A.; Pan, F.; Qi, R.; Roe, D. R.; Roitberg, A.; Sagui, C.; Schott-Verdugo, S.; Shen, J.; Simmerling, C. L.; Smith, J.; Salomon-Ferrer, R.; Swails, J.; Walker, R. C.; Wang, J.; Wei, H.; Wolf, R. M.; Wu, X.; Xiao, L.; York D. M.; Kollman, P. A., AMBER **2018**, University of California, San Francisco.

(11) Salomon-Ferrer, R.; Goetz, A. W.; Poole, D.; Le Grand, S.; Walker, R. C., Routine microsecond molecular dynamics simulations with AMBER - Part II: Particle Mesh Ewald. *J. Chem. Theory Comput.* **2013**, *9*, 3878-3888.

(12) Goetz, A. W.; Williamson, M. J.; Xu, D.; Poole, D.; Le Grand, S.; Walker, R. C., Routine microsecond molecular dynamics simulations with AMBER - Part I: Generalized Born. *J. Chem. Theory Comput*. **2012**, *8*, 1542-1555.

(13) Le Grand, S.; Goetz, A. W.; Walker, R. C., SPFP: Speed without compromise - a mixed precision model for GPU accelerated molecular dynamics simulations. *Comp. Phys. Comm*. **2013**, *184*, 374-380.

(14) Martínez, L.; Andrade, R.; Birgin, E. G.; Martínez, J. M., Packmol: A package for building initial configurations for molecular dynamics simulations. *J. Comput. Chem.* **2009**, *30(13)*, 2157-2164.

(15) Marson, D.; Posel, Z.; Posocco, P., Molecular features for probing small amphiphilic molecules with self-assembled monolayer-protected nanoparticles. *Langmuir* **2020**, 36, 5671–5679.

(16) You, W.; Tang, Z.; Chang, C. A. Potential mean force from umbrella sampling simulations: What can we learn and what is missed? *J. Chem. Theory Comput.* **2019**, 2433–2443.

(17) Grossfield, A., “WHAM: an implementation of the weighted histogram analysis method”, http://membrane.urmc.rochester.edu/content/wham/, version 2.0.11.

(18) Ferrenberg, A. M.; Swendsen, R. H., Optimized Monte Carlo data analysis. *Phys. Rev. Lett.***1989**, *63*, 1195–1198.

(19) Kumar, S.; Bouzida, D.; Swendsen, R. H.; Kollman, P. A.; Rosenberg, J. P., THE weighted histogram analysis method for free-energy calculations on biomolecules. I. The method. *J. Comput. Chem.* **1992**, *13*, 1011-1021.

(20) Kumar, S.; Rosenberg, J. M.; Bouzida, D.; Swendsen, R. H.; Kollman P. A. Multidimensional free-energy calculations using the weighted histogram analysis method. *J. Comput. Chem.* **1995**, *16*, 1399-1350.

(21) Doudou, S.; Burton, N. A.; Henchman, R. H. Standard free energy of binding from a one-dimensional potential of mean force. *J. Chem. Theory Comput.* **2009**, *5*, 909-918.

(22) Aho, N.; Groenhof, G.; Buslaev; P. Do all roads lead to Rome? Convergence issues in umbrella sampling simulations. *ChemRxiv* **2023**, doi: 10.26434/chemrxiv-2023-2pqls.

(23) Rad, N.; Sashuk, V., A light-gated regulation of the reaction site by a cucurbit[7]uril macrocycle. *Chem. Sci.* **2022,** *13*, 12440-12444.

(24) Guven, Z. P.; Silva, P. H. J.; Luo, Z.; Cendrowska, U. B.; Gasbarri, M.; Jones, S. T.; Stellacci, F., Synthesis and characterization of amphiphilic gold nanoparticles. *JoVE* **2019**, e58872.

(25) Sashuk, V.; Winkler, K.; Żywociński, A.; Wojciechowski, T.; Górecka, E.; Fiałkowski, M., Nanoparticles in a capillary trap: Dynamic self-assembly at fluid interfaces. *ACS Nano* **2013,** *7*, 8833-8839.

(26) Szewczyk, M.; Sobczak, G.; Sashuk, V., Photoswitchable catalysis by a small swinging molecule confined on the surface of a colloidal particle. *ACS Catal.* **2018,** *8*, 2810-2814.

(27) Martí-Centelles, V.; Piskorz, T. K.; Duarte, F., CageCavityCalc (C3): A computational tool for calculating and visualizing cavities in molecular cages. ChemRxiv **2024**, doi: 10.26434/chemrxiv-2024-fmlx0.
